# Supplementary material for: Learning from small data: Classifying sex from retinal images via deep learning
Source: PLoS One. 2023 Aug 3;18(8):e0289211. doi: 10.1371/journal.pone.0289211 (PMC10399793; doi:10.1371/journal.pone.0289211)
Supplement: S1 File — (ZIP) [file pone.0289211.s001.zip › berk2023learning_supp.pdf]

Supporting information: Learning from small data: Classifying sex from retinal images via deep learning

Aaron Berk[1*], Gulcenur Ozturan[2], Parsa Delavari[2] David Maberley[3], Özgür Yılmaz[4], Ipek Oruc[2]

1 Department of Mathematics & Statistics, McGill University, Montréal, Canada
2 Department of Ophthalmology and Visual Sciences, University of British Columbia, Vancouver, Canada
3 Department of Ophthalmology, University of Ottawa, Ottawa, Canada
4 Department of Mathematics, University of British Columbia, Vancouver, Canada

* aaron.berk@mcgill.ca

S1 Methods

Neural networks

A deep neural network is a highly parametrized function that is useful for general purpose function approximation. Suppose an unknown "ground truth" function $f : \mathbb{R}^d \to \{0, 1\}$ describes a labeling scheme for points $x \in \mathbb{R}^d$ (*i.e.*, $y = F(x)$ is the "label" for the point x). Further suppose one obtains several labeled realizations of data from this function: $\mathcal{S} := \{(x_1, y_1), \ldots, (x_n, y_n)\}$, where $y_i = F(x_i)$, $i \in [n]$. It is often desirable to be able to approximate the function F from the data $\mathcal{S}$, and in several complex, modern scenarios, the state-of-the-art choice is to train a deep neural network.

Let $\mathcal{H}$ denote a hypothesis class. For example, $\mathcal{H}$ may describe a particular deep neural network architecture (or set of architectures). The goal of training a deep neural network is, given $\mathcal{S} \subseteq \mathbb{R}^d$, to obtain $h \in \mathcal{H}, h : \mathbb{R}^d \to [0, 1]$ that approximates well the action of F on $x_i, i \in [n]$. In particular, given a convex function $\ell : [0, 1] \times \{0, 1\} \to \mathbb{R}_+$, find $h \in H$ so that $\ell(h(x), y) \ll 1$ for all $(x, y) \in \mathcal{S}$.

Metrics

Binary cross-entropy

Binary cross-entropy was the metric used to optimize the model — which is to say that the "misfit" that was approximately minimized during model training, between model predictions and the true labels associated to each image, was binary cross-entropy.

Receiver Operating Characteristic

Suppose a "ground truth" is given by the function $f : \mathcal{X} \to \{-1, 1\}$. Further, suppose an experimenter develops a hypothesis for the behaviour of f, in the form of a binary classifier $h : \mathcal{X} \to \{-1, 1\}$. Given a finite subset $\mathcal{S} \subseteq \mathcal{X}$, define the **true positive rate** and **false positive rate** of the hypothesis on the dataset $\mathcal{S}$ by, respectively,

$$\operatorname{tpr}(h, \mathcal{S}) := |P|^{-1} \sum_{x \in \mathcal{P}} \mathbb{1}(h(x) = 1) \qquad \operatorname{fpr}(h, \mathcal{S}) := |N|^{-1} \sum_{x \in \mathcal{N}} \mathbb{1}(h(x) = 1)$$

Now assume that h comes from a family of hypotheses, $h_\theta : \mathcal{X} \times \Theta \to \{0, 1\}$. For example, perhaps $h_\theta(\cdot) := \mathbb{1}\left(\tilde{h}(\cdot) > \theta\right)$ for some $\tilde{h} : \mathcal{X} \to [0, 1]$, and $\Theta = [0, 1]$. In this case, $\tilde{h}$ outputs a *score* for the point x, and θ acts as a *threshold*. By a mild abuse of notation, we can define the true positive rate and false positive rate as functions of the threshold θ:

$$\operatorname{tpr}(\theta) := |P|^{-1} \sum_{x \in \mathcal{P}} \mathbb{1}(h_\theta(x) = 1) \qquad \operatorname{fpr}(\theta) := |N|^{-1} \sum_{x \in \mathcal{N}} \mathbb{1}(h_\theta(x) = 1)$$

The **receiver operating characteristic** (ROC) of the function h_θ on the dataset $\mathcal{S}$ is given as the

$$\operatorname{ROC}(h; \mathcal{S}, \Theta) := \{(\operatorname{fpr}(\theta), \operatorname{tpr}(\theta)) : \theta \in \Theta\}$$

We can view $\operatorname{ROC}(h; \mathcal{S}, \Theta)$ as a graph, with the true positive rate changing as a function of the false positive rate. If h is a good model for f, then the true positive rate and true negative rate are high. Now, $\operatorname{fpr} = 1 - \operatorname{tnr}$, where tnr is the true negative rate. In particular, the true positive rate is high and the false positive rate is close to zero. Also observe that tpr is a monotonically non-decreasing function of fpr. Thus, if h is a good model for f, then the area under the curve of the ROC should be large. To measure this area, we simply integrate as follows:

$$\operatorname{AUC}(h; \mathcal{S}) := \int_0^1 \operatorname{tpr}(\operatorname{fpr}^{-1}(\theta))\, \mathrm{d}\theta. \tag{1}$$

Network architecture

The network architecture used in this work is modified from a deep residual network, ResNet-152, originally developed in [1]. This network is composed of *residual units* $\mathcal{H}(x)$, which resemble the following:

$$\mathcal{H}(x) := x + \mathcal{F}(x), \quad \text{where} \quad \mathcal{F}(x) := W_2 \sigma(W_1 x + b_1) + b_2.$$

One residual unit may be composed with a subsequent residual unit as $\tilde{\mathcal{H}}(x) := \mathcal{H}^2(\sigma(\mathcal{H}^1(x)))$, where, as above, σ is an activation function. In the implementation, there are typically more than two matrices W_i in each unit; the matrices W_i typically correspond to a set of convolutions of a fixed filter size (*e.g.,* 64 convolution filters of size 3×3); and the activation function σ is typically `ReLU`. Moreover, the network may not be entirely composed of residual units: portions resembling more classical architectures may be used as well. For example, it is common to append one or more fully connected layers to a residual network: if f_1, f_2 are fully connected layers, so that $f_i(x) = \tilde{W}_i x + \tilde{b}$ for $i \in [3]$ where W_i and b_i are dense, then it is common to construct a network resembling

$$\tilde{\mathcal{H}}(x) := (f_3 \circ f_2 \circ f_1 \circ \mathcal{H}^k \circ \sigma \circ \cdots \circ \mathcal{H}^2 \circ \sigma \circ \mathcal{H}^1)(x).$$

For full implementation details of ResNet-152 we refer the reader to [1] as well as the PyTorch [2] source code for the `ResNet` class, available in `torchvision.models.resnet` available on GitHub.

The network architecture was modified from that of [1] by removing the final layer of the network and appending 2 fully connected layers, with Dropout. The new layers of the network were randomly initialized according to PyTorch `v1.01` [2] default settings.

S1 Choice of loss

While training-time binary cross-entropy (BCE) loss monotonically decreases on the training partition, we observed that validation BCE loss increased for a majority of the epochs, despite continued improvements on the accuracy and AUC metrics (see Figures 1–3). To explain this behavior, we examined the output probabilities of the model throughout the training epochs on ODIR-N. As shown in Figure S1, training samples are not separable during early epochs but become so: as training progresses the histograms eventually have disjoint support. Ultimately, training samples are completely separable and the model returns only probabilities close to zero or one, which means it is *confident* in its classifications on the training set.

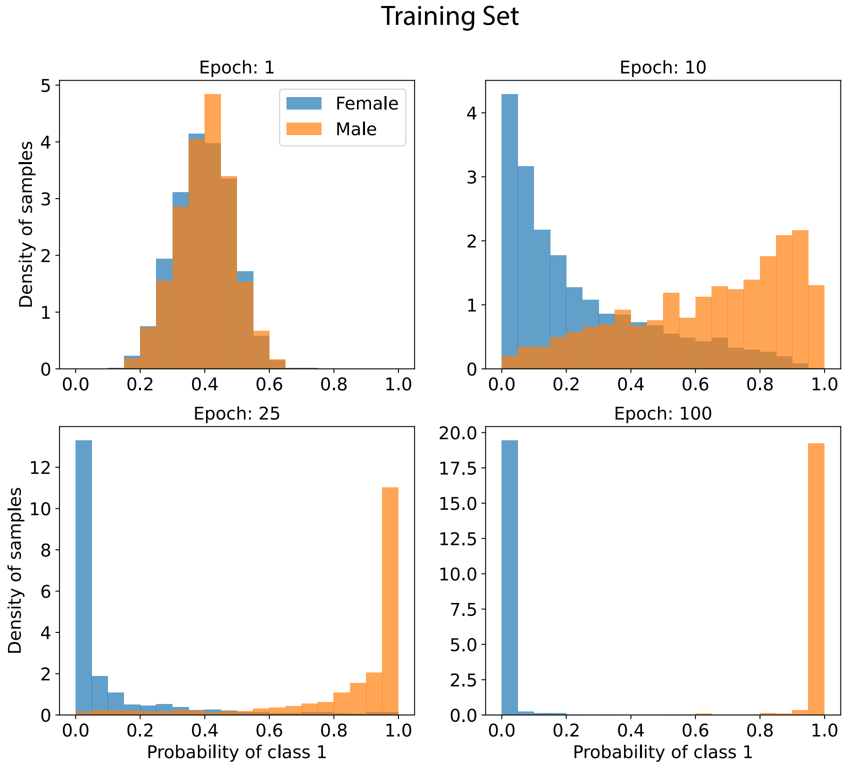

Fig S1. The histogram of predicted probabilities for training samples over training epochs: blue denotes female samples (class 0), orange denotes male samples (class 1).

Although the model's performance is not as high and the two histograms overlap, the model insists on confident decisions on the validation partition (see Figure S2). Indeed, at the end of the training process, the majority of the predicted probabilities are close to zero or one. However, in contrast to the training set, some of the probabilities are predicted incorrectly. By design, confident incorrect classifications incur exponentially larger BCE loss. We argue, thereby, that the "disproportionate" loss incurred by confident incorrect classifications explains the training-time behavior of the BCE loss on the validation partition.

In Figure S3a, BCE loss is plotted as a function of predicted probability when the true label is one (*i.e.,* the model's *belief* that the true label is one). When the true label is predicted by the model with probability one, the loss is zero, and as the probability

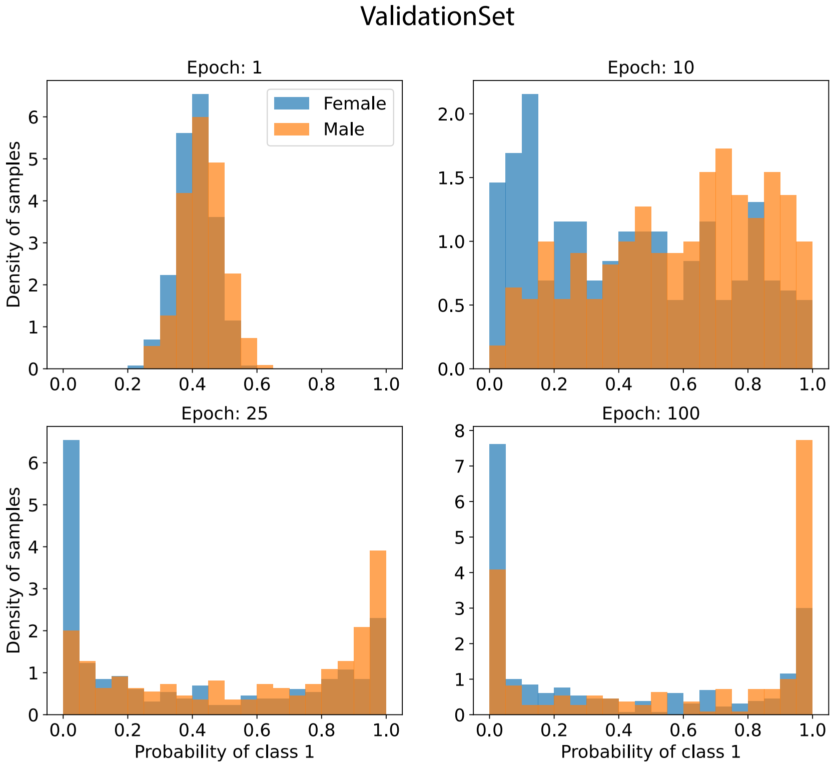

Fig S2. Histograms of predicted probabilities for validation samples, stratified by training epoch. Blue denotes female samples (class 0), orange male (class 1).

decreases, the loss increases exponentially. The model's decision boundary is at 0.5 probability, at which point, the predicted label switches. We can see that loss for probabilities higher than this threshold (where the model predicts the true label) is low and negligible. However, by definition of BCE, a much higher penalty is incurred for incorrectly predicted probabilities, especially probabilities around zero at which the loss function approaches infinity. Therefore, although the two histograms in Figure S2 separate during the training epochs, the incorrect but confident predictions contribute large loss. This increase in loss is not compensated by additional correct predictions in progressive epochs; thus, we observe that loss increases for the validation set during training.

To test this hypothesis, we trained a model on ODIR-N once with BCE loss and once with a "balanced" cosine loss, as a comparison. When the true label is one, the balanced loss is defined by $\text{loss}(p \mid y = 1) := 1 + \cos(\pi p)$, as represented in Figure S3b; analogously, $\text{loss}(p \mid y = 0) := 1 - \cos(\pi p)$. The methodological details of these supplementary experiments are identical to those of the main experiments reported in Results. The choice of the new loss function was arbitrary in the sense that the only constraint be a balanced change in penalty with respect to "confidence" for incorrect and correct decisions. In other words, an increase in loss due to increased confidence on an incorrect classification is bounded and comparable to a decrease in loss when incorrect decisions are replaced with correct ones. As shown in Figure S4, validation loss decreased during training with the new bounded loss, in contrast to training with BCE loss. This occurred even though the model still learned confident incorrect

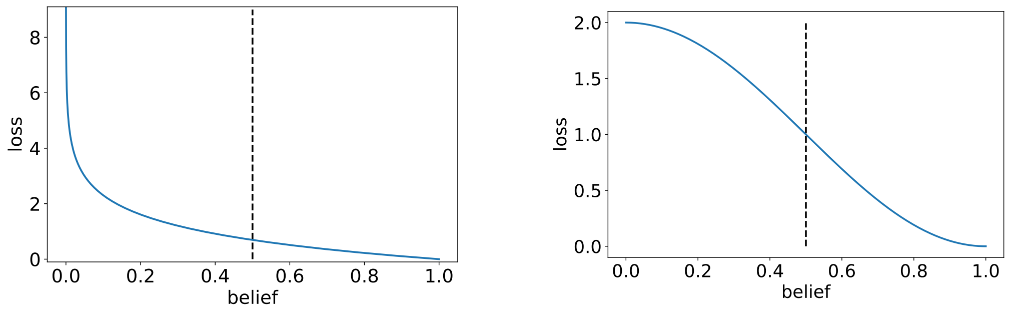

(a) binary cross-entropy loss

(b) balanced loss

Fig S3. For each loss function, loss *vs.* belief for class 1 when the true label is 1.

classifications on the validation data (see Figure S5). In conclusion, this experiment illustrates that the increasing pattern of validation loss observed during training can be remedied by replacing BCE loss with another.

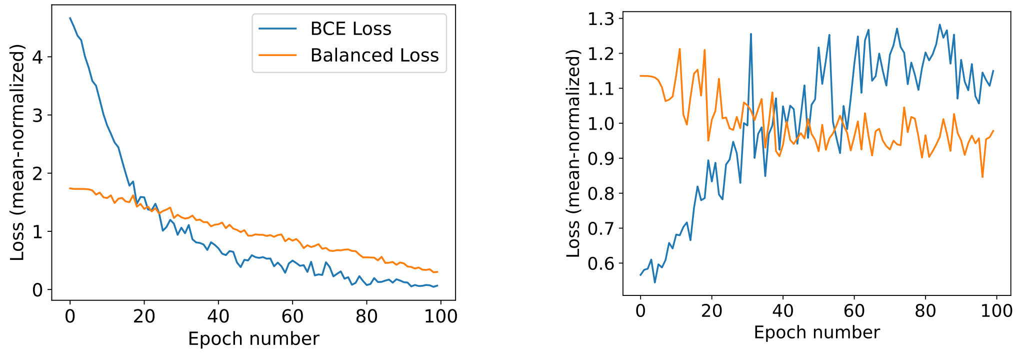

(a) training

(b) validation

Fig S4. BCE loss and the balanced loss on the training set (left panel) and validation set (right panel) plotted as a function of epochs.

S1 Bootstrap method

For a more thorough background on ideas related to and making use of bootstrapping, refer to [3, § 6.2], from which this section draws its main ideas.

Suppose that $x = (x_1, \dots, x_n)$ is a random sample, with $x_i \sim \mathcal{F}, i \in [n]$, where $\mathcal{F}$ is a distribution. Further suppose we wish to estimate a parameter of interest $\theta = t(\mathcal{F})$ using the sample x, where t is some mapping yielding the "true" quantity θ corresponding to the distribution $\mathcal{F}$. In order to estimate θ, suppose we calculate $\hat{\theta} := s(x)$ for a mapping s that takes the random sample x to a quantity $\hat{\theta}$. For example, one might have $s(x) := t(\hat{\mathcal{F}})$ where $\hat{\mathcal{F}}$ is the empirical distribution constructed from x:

$$\hat{\mathcal{F}} := \sum_{i \in [n]} \delta_{x_i}.$$

In the present setting, we might have $x \in (\{0, 1\} \times [0, 1])^n$ with $x_i, i \in [n]$ being the label for image i and the classifier's probability that image i is class "male". Thus, t gives the AUC for the true distribution of images $\mathcal{F}$, and we approximate the true distribution by computing $s(x) := \mathrm{AUC}(x)$ where AUC is given in (1).

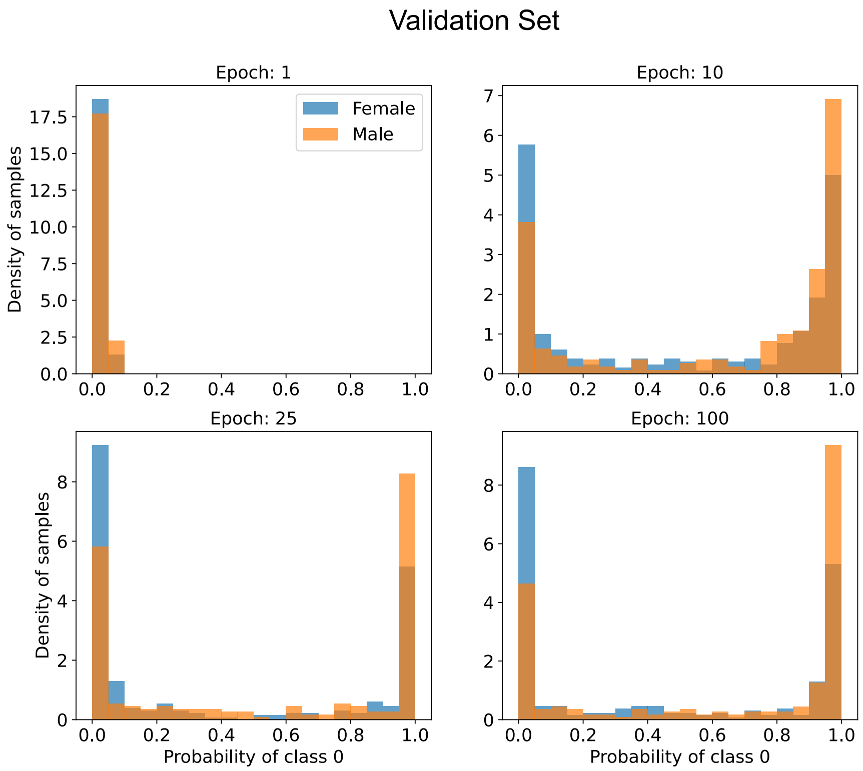

Fig S5. Histograms of model beliefs for validation data when trained with the balanced loss. Histograms are stratified by training epochs. Blue denotes female samples (class 0), orange denotes male samples (class 1). This graphic is a direct analogue of Figure S2 for the balanced loss.

Define a bootstrap sample of size n by $x^* := (x_1^*, \ldots, x_n^*)$ where $x_i^* \sim \hat{\mathcal{F}}$ are sampled with replacement from $\hat{\mathcal{F}}$. x^* is a randomized, resampled version of x. The idea of bootstrapping is to evaluate the quality of $\hat{\theta}$ by analyzing the distribution of $\hat{\theta}^* := s(x^*)$.

It is worth noting that the bootstrap estimate of the standard error of a statistic $\hat{\theta}$ is a plug-in estimate. Namely, the ideal bootstrap estimate of the standard error of $\hat{\theta}$ is $\mathrm{se}_{\hat{\mathcal{F}}}(\hat{\theta}^*)$. As a recipe for the so-called non-parametric bootstrap for the standard error of a statistic $\hat{\theta}$:

1. Generate $(x^{*(b)})_{b=1}^B$ where each $x^{*(b)}$ has n samples drawn from $\hat{\mathcal{F}}$;
2. Compute $\hat{\theta}^*(b) := s(x^{*(b)}), b \in [B]$;
3. Estimate $\mathrm{se}_{\mathcal{F}}(\hat{\theta}) \approx \widehat{\mathrm{se}}_B$

Note: in this work, we do not estimate the standard error, but rather quantiles. See Efron and Tibshirani [3], Ghosh *et al.* [4], Babu [5] for more on bootstrapping and bootstrapping quantiles, in particular.

S1 Parameter values

Runs D1–5

Below, we list the parameter values used for the sex classification task using the DOVS-i database. The same parameter settings were used for runs D1 through D5.

Optimizer ::

- **method:** SGD
- **learning rate:** 10^{-3}
- **batch size:** 16
- **weight decay:** 10^{-3}
- **acceleration:** Nesterov
- **momentum:** 0.9
- **annealing:** `ExponentialLR`(0.99)

Criterion ::

- **Max epochs:** 1000
- **early stopping:** validation AUC
- **loss:** binary cross-entropy
- **class weights (F, M):** $(0.98, 1.02)$

Network ::

- **base network:** ResNet-152
- **hidden layers:** 2048
- **dropout probability:** 0.5

Transforms ::

Train ::

```
ColorJitter(
    brightness=[0.95, 1.05],
    contrast=[0.95, 1.05],
    saturation=[0.95, 1.05],
    hue=[-0.05, 0.05]
),
RandomHorizontalFlip(p=0.5),
RandomVerticalFlip(p=0.5),
Normalize(
    mean=[0.485, 0.456, 0.406],
    std=[0.229, 0.224, 0.225]
)
```

Val & Test ::

```
Normalize(
    mean=[0.485, 0.456, 0.406],
    std=[0.229, 0.224, 0.225]
)
```

Runs N1–6

Below are the parameter values used in the sex classification task trained on the ODIR-N database. The same parameter settings were used for runs N1 through N6, except for the omission of channel normalization in the image preprocessing transform for runs N1 through N3. In runs N1 through N3, the preprocessed images were not normalized (using `transforms.Normalize`) before being fed into the network. This is reflected in the description below. In runs N4 through N6, normalization of the channels was applied (using `transforms.Normalize`) with the same mean and variance as that for DOVS-i and ODIR-C (*cf.* below). When the channel normalization was used, it was applied to training, validation and test images alike, as the final transform before images were fed into the network.

Optimizer ::

- **method:** SGD
- **learning rate:** 10^{-3}
- **batch size:** 16
- **weight decay:** 10^{-3}
- **acceleration:** Nesterov
- **momentum:** 0.9
- **annealing:** `ExponentialLR`(0.99)

Criterion ::

- **Max epochs:** 1000
- **early stopping:** validation AUC
- **loss:** binary cross-entropy
- **class weights (F, M):** $(0.91, 1.11)$

Network ::

- **base network:** ResNet-152
- **hidden layers:** 1024
- **dropout probability:** 0.3

Transforms ::

Train ::

```
ColorJitter(
    brightness=[0.95, 1.05],
    contrast=[0.95, 1.05],
    saturation=[0.95, 1.05],
    hue=[-0.05, 0.05]
),
RandomHorizontalFlip(p=0.5),
RandomVerticalFlip(p=0.5)
```

Runs C1–6

These are the parameter values for the sex classification task trained using the ODIR-C database. The same parameters were used for runs C1 through C6.

Optimizer ::

- **method:** SGD
- **learning rate:** 10^{-3}
- **batch size:** 16
- **weight decay:** 10^{-3}
- **acceleration:** Nesterov
- **momentum:** 0.9
- **annealing:** `ExponentialLR`(0.99)

Criterion ::

- **Max epochs:** 1000
- **early stopping:** validation AUC
- **loss:** binary cross-entropy
- **class weights (F, M):** $(0.92, 1.10)$

Network ::

- **base network:** ResNet-152
- **hidden layers:** 2048
- **dropout probability:** 0.5

Transforms ::

Train ::

```
ColorJitter(
    brightness=[0.95, 1.05],
    contrast=[0.95, 1.05],
    saturation=[0.95, 1.05],
    hue=[-0.05, 0.05]
),
RandomHorizontalFlip(p=0.5),
RandomVerticalFlip(p=0.5),
Normalize(
    mean=[0.485, 0.456, 0.406],
    std=[0.229, 0.224, 0.225]
)
```

Val & Test ::

```
Normalize(
    mean=[0.485, 0.456, 0.406],
    std=[0.229, 0.224, 0.225]
)
```

Runs E1–20

In this section we include the parameter values used in the sex classification task that used an ensemble of 10 ResNet-152 models trained using the DOVS-ii database. The same parameters were used for runs E1 through E20.

Optimizer ::

method: SGD
learning rate: 10^{-3}
batch size: 16
weight decay: 10^{-3}
acceleration: Nesterov
momentum: 0.9
annealing: `ExponentialLR`(0.99)

Criterion ::

Max epochs: 1000
early stopping: validation AUC
loss: binary cross-entropy
class weights (F, M): $(0.98, 1.02)$

Network ::

base network: ResNet-152
hidden layers: 2048
dropout probability: 0.5

Transforms ::

Train ::

```
ColorJitter(
    brightness=[0.95, 1.05],
    contrast=[0.95, 1.05],
    saturation=[0.95, 1.05],
    hue=[-0.05, 0.05]
),
RandomHorizontalFlip(p=0.5),
RandomVerticalFlip(p=0.5),
Normalize(
    mean=[0.485, 0.456, 0.406],
    std=[0.229, 0.224, 0.225]
)
```

Val & Test ::

```
Normalize(
    mean=[0.485, 0.456, 0.406],
    std=[0.229, 0.224, 0.225]
)
```

S1 Data statistics

DOVS-i & DOVS-ii

The original image sizes were 2392×2048. The images were resized to 224×224, an image size that has been used for other image classification tasks [1, 6]. To perform the resizing, the images were first resized to the nearest power of 2 using a discrete Haar wavelet transform [7], and then resized to 224×224 using bilinear intepolation *via* a standard Python package for image manipulation tasks [8].

We note here that the DOVS-ii database is a superset of the DOVS-i database — a result of an additional round of data retrieval from the VGH servers. Properties of and preprocessing for images in DOVS-i is the same as that for DOVS-ii.

Patient statistics are summarized in Figure S6 and Table 1. Histograms for age data appear in Figure S6 for DOVS-i (top) and DOVS-ii (bottom). Descriptive statistics stratified by patient age and sex appear in Table 1a for DOVS-i and Table 1b for DOVS-ii.

ODIR

The ODIR database was curated by hosts of an online competition for machine learning on ocular disease [9]. This database contains 7000 annotated images from 3500 patients — a left/right pair of images for each patient in the database.

DOVS-i

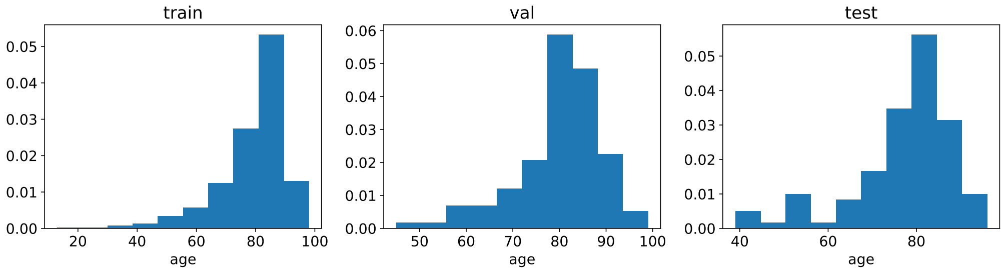

DOVS-ii

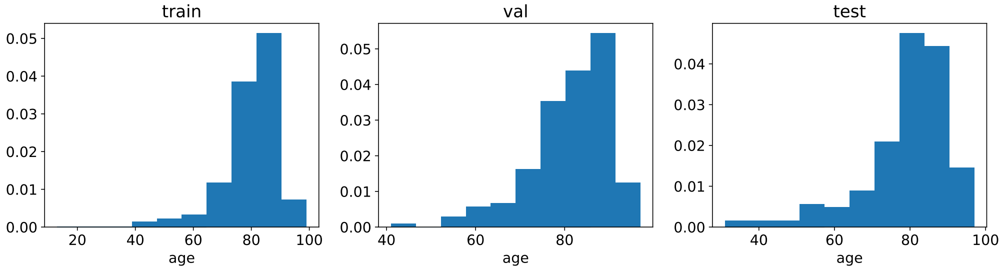

Fig S6. DOVS-i and DOVS-ii patient age statistics: phase-stratified density-style histograms representing the patient age distributions for each dataset.

ODIR-N

The ODIR-N variant of the ODIR database was curated by us. It was obtained by subsampling the ODIR database, retaining only those eye images that were "normal" (*i.e.,* no adverse health condition annotation was present). After subsampling of the database, the images were cropped to a bounding box about the fundus image and downsampled to a size of 224×224 as described in DOVS-i & DOVS-ii. Finally, the colour channels of the images were adjusted using CLAHE [10]. We coin as ODIR-N the resulting dataset of 3098 images from 1959 individuals. The patients in this database were randomly partitioned into three datasets for training, validation and test sets, comprising 70%, 15% and 15% of the patients, respectively (*cf.* Table 1c). See Table 1c for patient age statistics stratified by phase, with and without stratification by patient sex.

ODIR-C

The ODIR-C database is a subset of the ODIR-N database, obtained by admitting or omitting images through a quality vetting procedure. The train/val/test partitions of the ODIR-C images are different than those of the ODIR-N images. For example, it is very likely that there exist two images in the train set of ODIR-N such that one resides in the train set of ODIR-C and the other in the test set of ODIR-C. However, the proportions in each partition are the same (*cf.* Table 1d). See Table 1d for patient age statistics stratified by phase, with and without stratification by patient sex.

We now describe the vetting procedure, which effectively follows a combined ruleset of those used by Gulshan *et al.* [11] and Ting *et al.* [12]. Specifically, images from ODIR-N are admitted/omitted on the basis of the following items.

1. Illumination: Is the image too dark, or too light? Are there dark areas or washed-out areas?

	train			val			test		
	F	M	∪	F	M	∪	F	M	∪
min	24	13	13	55	45	45	53	39	39
max	98	97	98	96	99	99	95	96	96
mean	79.59	78.06	78.84	80.34	79.73	80.05	79.44	74.54	77.04
st.dev.	11.76	10.96	11.40	8.05	10.31	9.18	8.88	13.31	11.51

(a) DOVS-i age statistics.

	train			val			test		
	F	M	∪	F	M	∪	F	M	∪
min	24	13	13	55	41	41	31	37	31
max	98	99	99	97	95	97	97	97	97
mean	80.17	79.18	79.68	82.93	79.36	81.17	79.58	78.60	79.10
st.dev.	9.60	9.65	9.63	8.17	9.73	9.14	11.60	11.07	11.34

(b) DOVS-ii age statistics.

	train			val			test		
	F	M	∪	F	M	∪	F	M	∪
min	1	17	1	28	25	25	22	15	15
max	89	89	89	85	87	87	88	91	91
mean	58.6	56.4	57.4	59.3	56.1	57.6	58.8	55.7	57.1
st.dev.	11.5	11.0	11.3	10.6	11.6	11.3	11.7	11.5	11.6

(c) ODIR-N age statistics.

	train			val			test		
	F	M	∪	F	M	∪	F	M	∪
min	1	15	1	1	17	1	26	31	26
max	87	87	87	87	82	87	82	89	89
mean	58.1	56.0	57.0	56.4	54.2	55.2	58.9	55.4	57.0
st.dev.	10.9	11.1	11.0	12.7	10.5	11.6	10.2	10.9	10.7

(d) ODIR-C age statistics.

Table 1. Patient age statistics: phase- and sex-stratified summary statistics for patient age within each dataset. The "F" columns correspond to statistics for female patients; "M", male patients; ∪, no stratification by sex.

2. Image field definition: Does the primary field include the entire optic nerve head and macula?
3. Artifacts: Is the image sufficiently free of artifacts (*e.g.,* dust spots, arc defects, and eyelash images)?
4. Validity: Is the image a valid "retinal image"?
5. Compositeness: Is the image acquired from a single capture event with normal angle of view which provides a 30 to 50 degree image or was it composited?

In the case of 4, a retinal image may be *invalid*, for example, if there is media opacity (such as cataracts). This exclusion is consistent with Ting *et al.* [12]. Regarding 5, composite images were excluded from ODIR-C.

S1 GGCAM plots

new

In this section we present the indivdiual GGCAM and GGCAM-amp images used to create the aggregate visualizations presented in Visual explanations for DOVS-ii classifiers. Those for the "Female" eyes are in Figure S7; those for "Male" eyes are in Figure S8. Each model's classification for the given fundus photo is displayed in each corresponding column heading.

GGCAM model development statistics

Here we include the model development statistics for the models used to produce the GGCAM graphics in this section and Visual explanations for DOVS-ii classifiers. As stated there, the procedure used to train and validate the models was identical to the one described for E1–E10. Thus, early stopping was again used to select the best epoch for each run according to the validation AUC. The selected epoch and the validation AUC, as well as the test AUC (scored on the DOVS-ii test partition), are presented in Table 2. As is probable, in view of the tests for confidence performed in Table 3, the validation and test AUC scores of these new six models are near to those of E1–E10. We explicitly note here the assumption that it is reasonable to expect the GGCAM results for these six models to similar to the ones one might have obtained using E1–E10.

Model	Epoch	AUC	
		val	test
G1	44	0.759	0.679
G2	18	0.758	0.700
G3	19	0.750	0.687
G4	14	0.732	0.702
G5	10	0.743	0.698
G6	13	0.750	0.695

Table 2. Model development statistics for six DOVS-ii models G1–G6 trained for the purpose of Guided Grad-CAM visualizations. Columns from left to right: model name, epoch selected epoch by validation AUC, validation AUC and test AUC.

GGCAM normalization

The colour-range normalization for the GGCAMs in the leftmost column of each subfigure in Figure 6 is equal to the largest absolute value in each row of Table 3 (as described in Visual explanations for classification performance). For example, in the case of F227_R, if a channel at a pixel is plotted with a value of 1, then corresponding Guided Grad-CAM value in that channel is equal to 0.33.

base image	min	max
F227_R	-0.30	0.33
F22_L	-0.47	0.28
M218_L	-1.28	1.35
M273_R	-0.72	1.56

Table 3. Guided Grad-CAM gradient extrema

References

1. He K, Zhang X, Ren S, Sun J. Deep residual learning for image recognition. In Proceedings of the IEEE Conference on Computer Vision and Pattern Recognition. p. 770–778.

2. Paszke A, Gross S, Chintala S, Chanan G, Yang E, DeVito Z, Lin Z, Desmaison A, Antiga L, Lerer A. Automatic differentiation in PyTorch. In NIPS Autodiff Workshop.

3. Efron B, Tibshirani RJ. An introduction to the bootstrap. CRC press. 1994.

4. Ghosh M, Parr WC, Singh K, Babu GJ. A note on bootstrapping the sample median. The Annals of Statistics. 1984. 12(3):1130–1135.

5. Babu GJ. A note on bootstrapping the variance of sample quantile. Annals of the Institute of Statistical Mathematics. 1986. 38(3):439–443.

6. Howard AG, Zhu M, Chen B, Kalenichenko D, Wang W, Weyand T, Andreetto M, Adam H. Mobilenets: Efficient convolutional neural networks for mobile vision applications. arXiv preprint arXiv:1704.04861. 2017.

7. Lee G, Gommers R, Waselewski F, Wohlfahrt K, O'Leary A. Pywavelets: a python package for wavelet analysis. Journal of Open Source Software. 2019. 4(36):1237.

8. Clark A. Pillow (pil fork) documentation. 2015. https://buildmedia.readthedocs.org/media/pdf/pillow/latest/pillow.pdf

9. Shanggong Medical Technology Co Ltd. Peking university international competition on ocular disease intelligent recognition. https://odir2019.grand-challenge.org. 2019.

10. Buslaev A, Iglovikov VI, Khvedchenya E, Parinov A, Druzhinin M, Kalinin AA. Albumentations: fast and flexible image augmentations. Information. 2020. 11(2):125.

11. Gulshan V, Peng L, Coram M, Stumpe MC, Wu D, Narayanaswamy A, Venugopalan S, Widner K, Madams T, Cuadros J, et al.. Development and validation of a deep learning algorithm for detection of diabetic retinopathy in retinal fundus photographs. Jama. 2016. 316(22):2402–2410.

12. Ting DSW, Cheung CYL, Lim G, Tan GSW, Quang ND, Gan A, Hamzah H, Garcia-Franco R, San Yeo IY, Lee SY, et al.. Development and validation of a deep learning system for diabetic retinopathy and related eye diseases using retinal images from multiethnic populations with diabetes. Jama. 2017. 318(22):2211–2223.

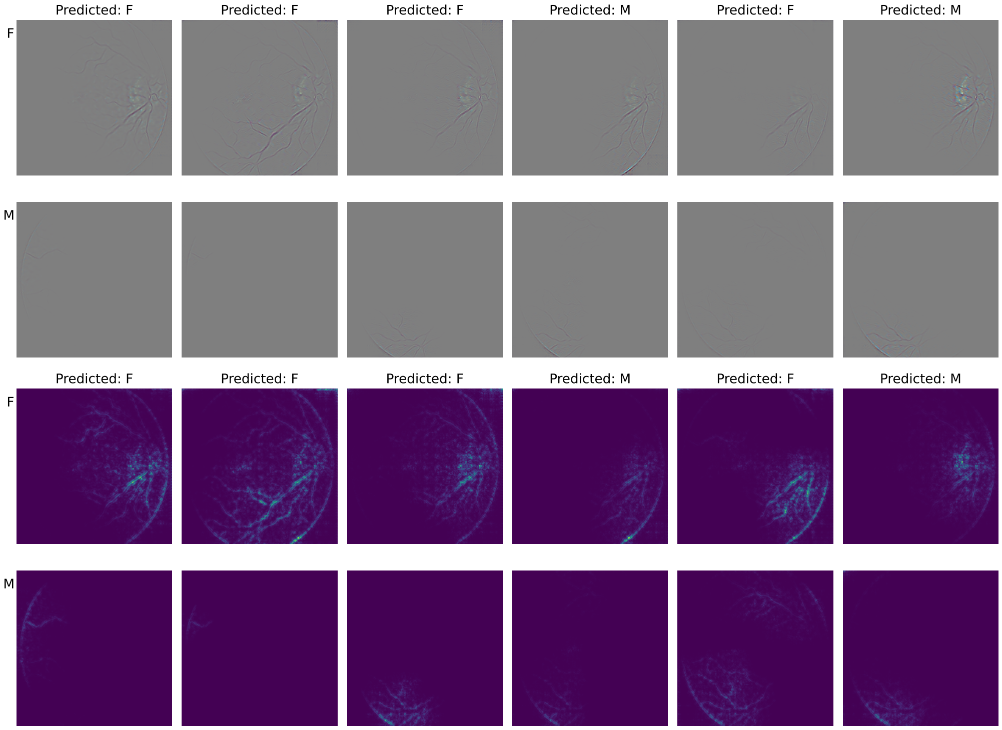

(a) F227_R

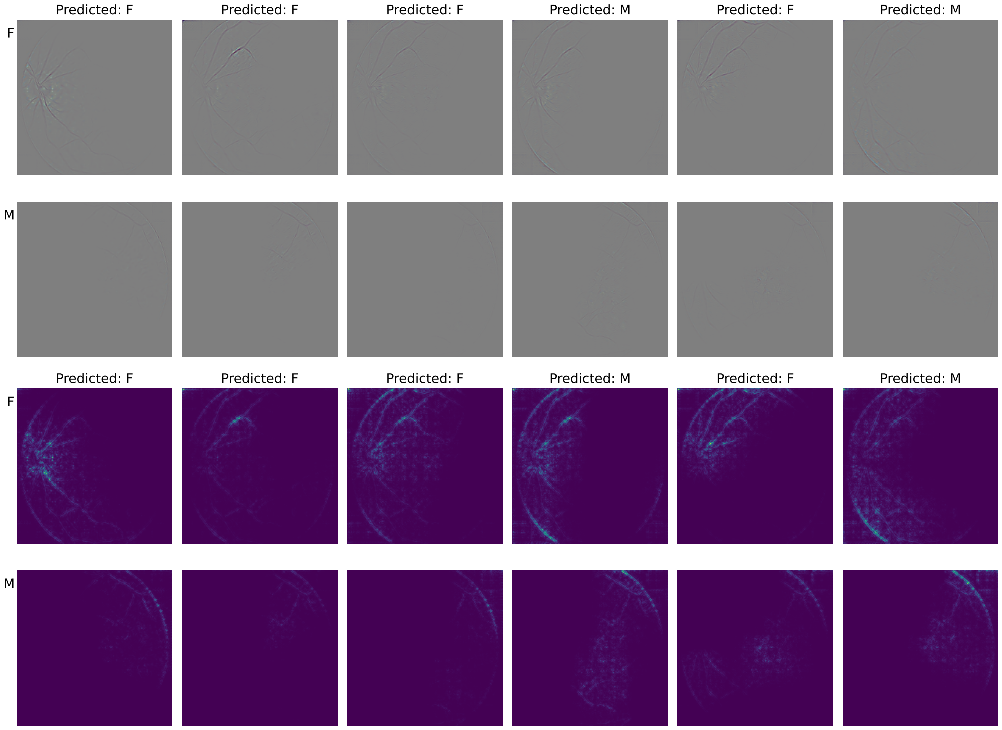

(b) F22_L

Fig S7. Individual Guided Grad-CAM and Guided Grad-CAM-amp images for two "Female" fundus photos.

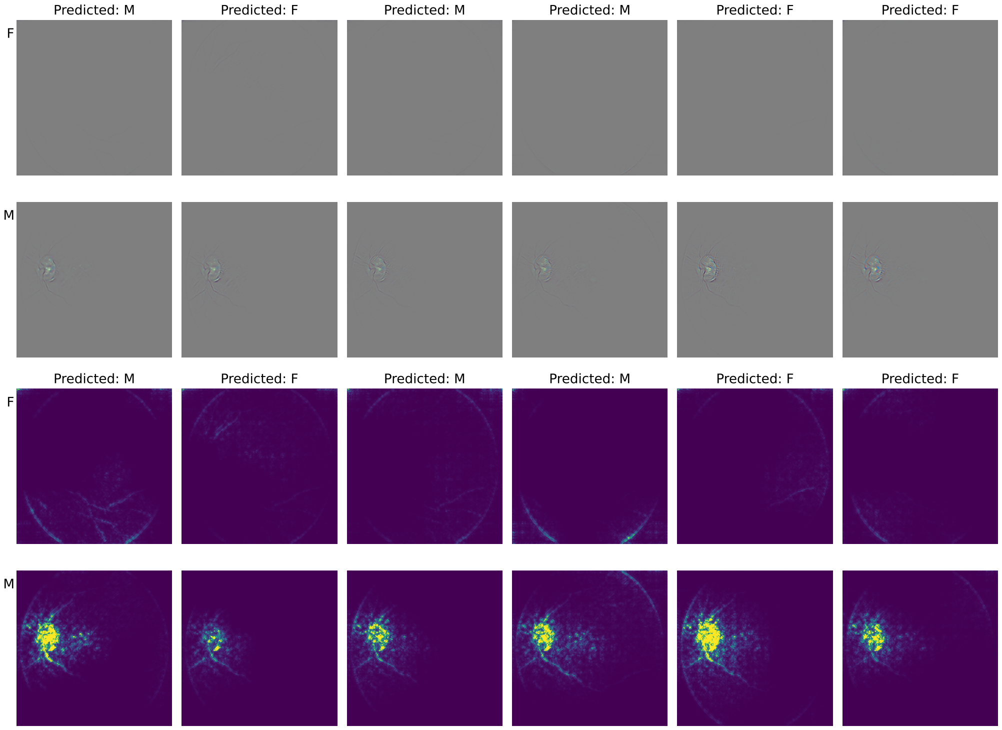

(a) M218_L

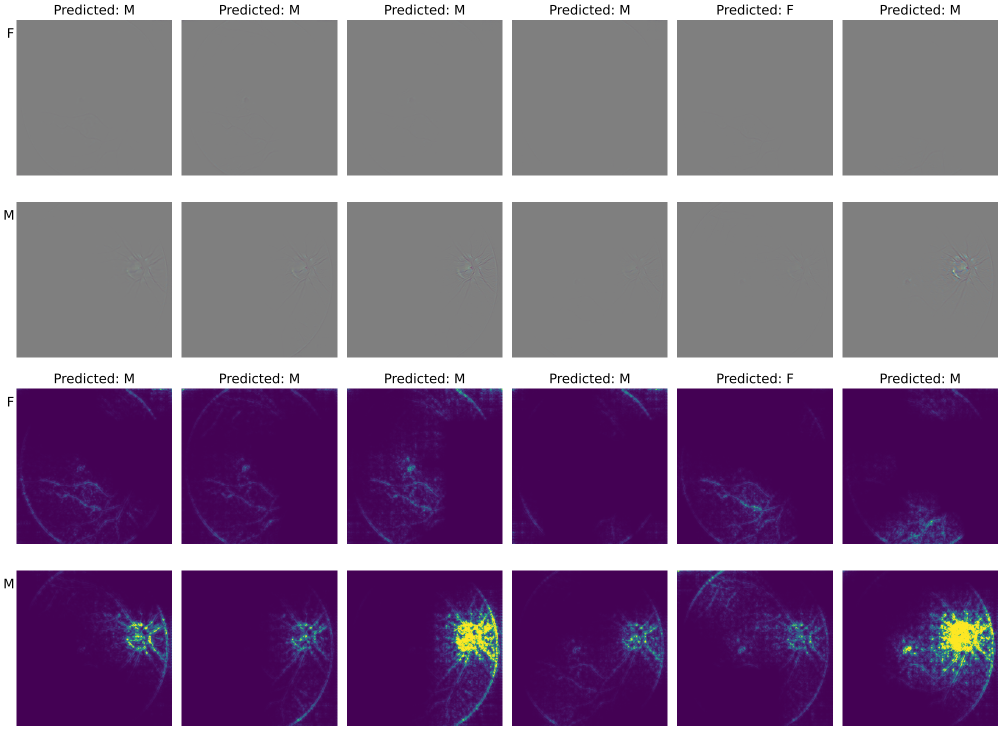

(b) M273_R

Fig S8. Individual Guided Grad-CAM and Guided Grad-CAM-amp images for two "Male" fundus photos.
