# Supplementary figures and images for: Learning from small data: Classifying sex from retinal images via deep learning
Source: PLoS One. 2023 Aug 3;18(8):e0289211. doi: 10.1371/journal.pone.0289211 (PMC10399793; doi:10.1371/journal.pone.0289211)

# Training Set

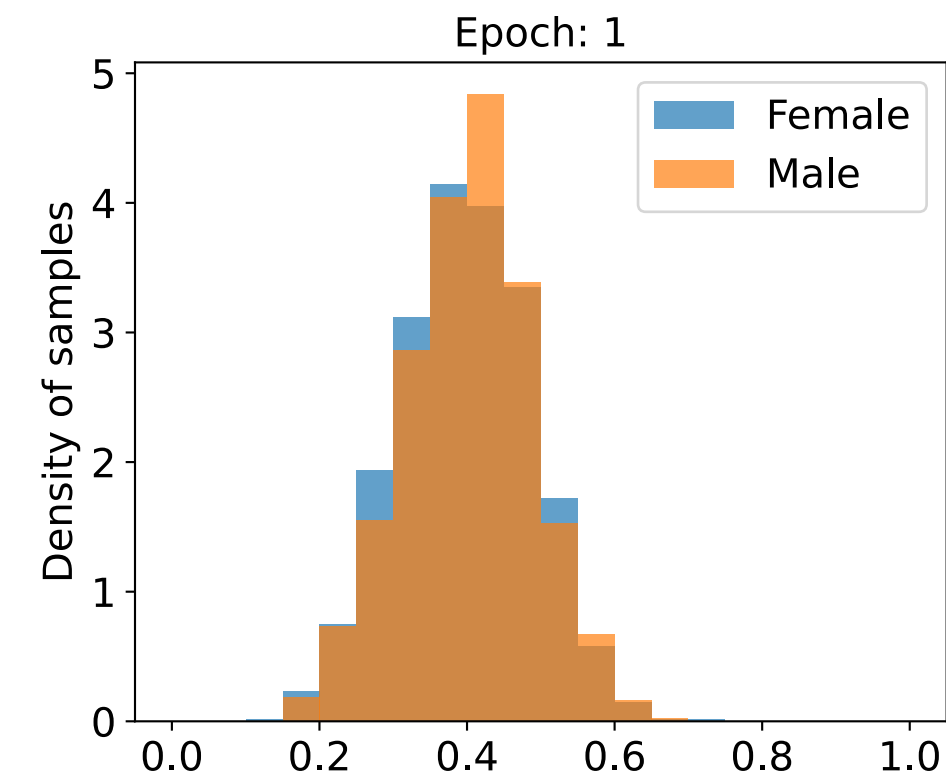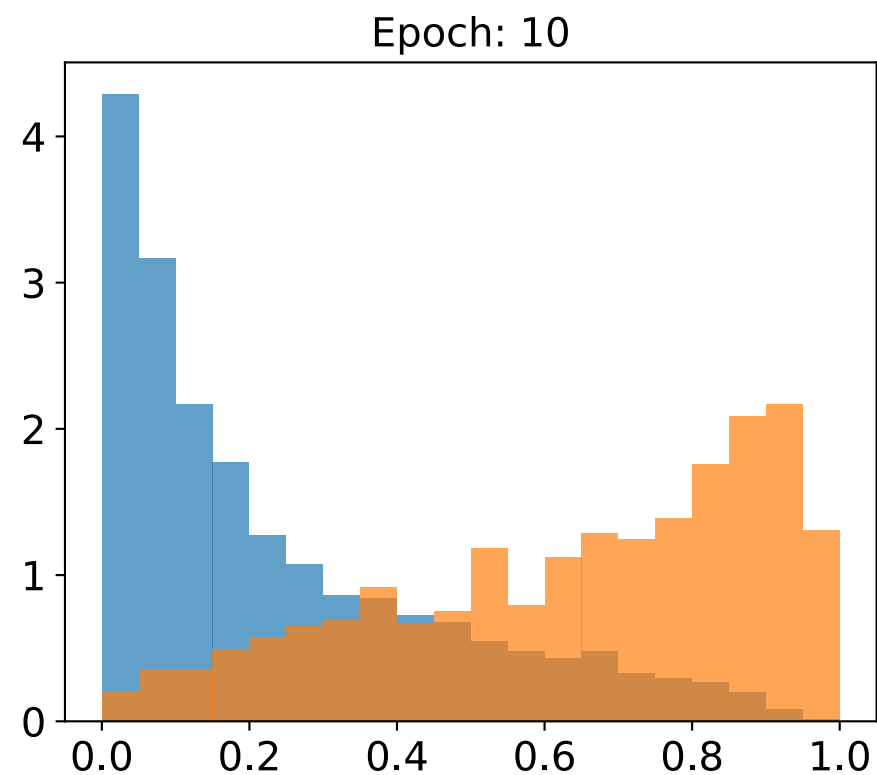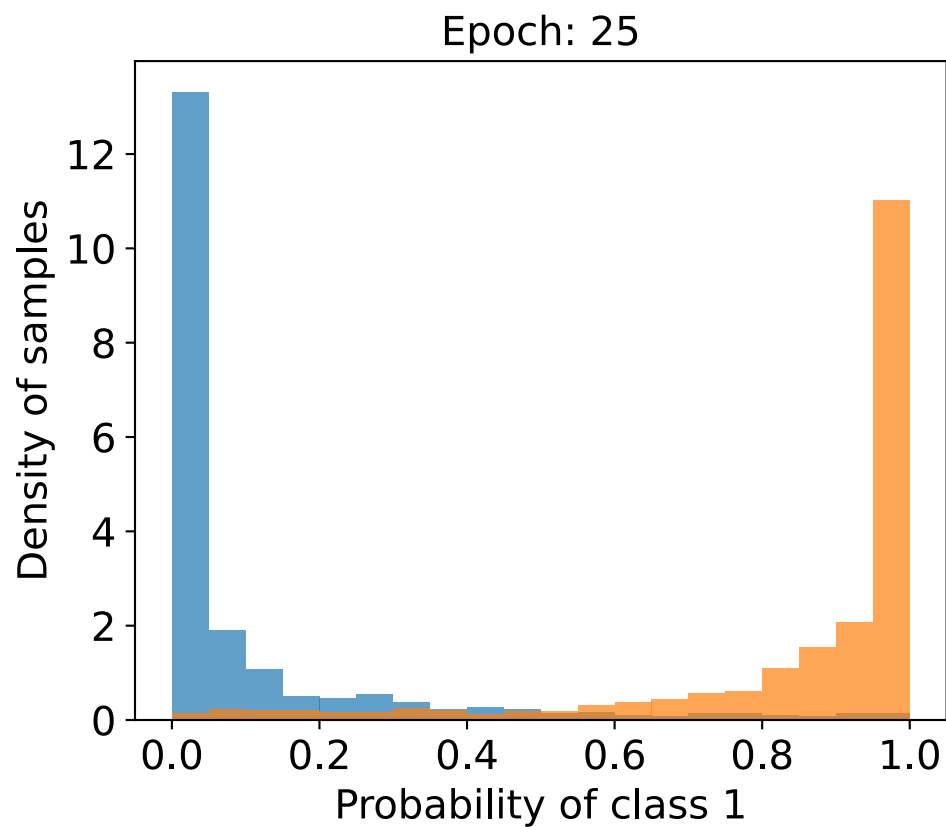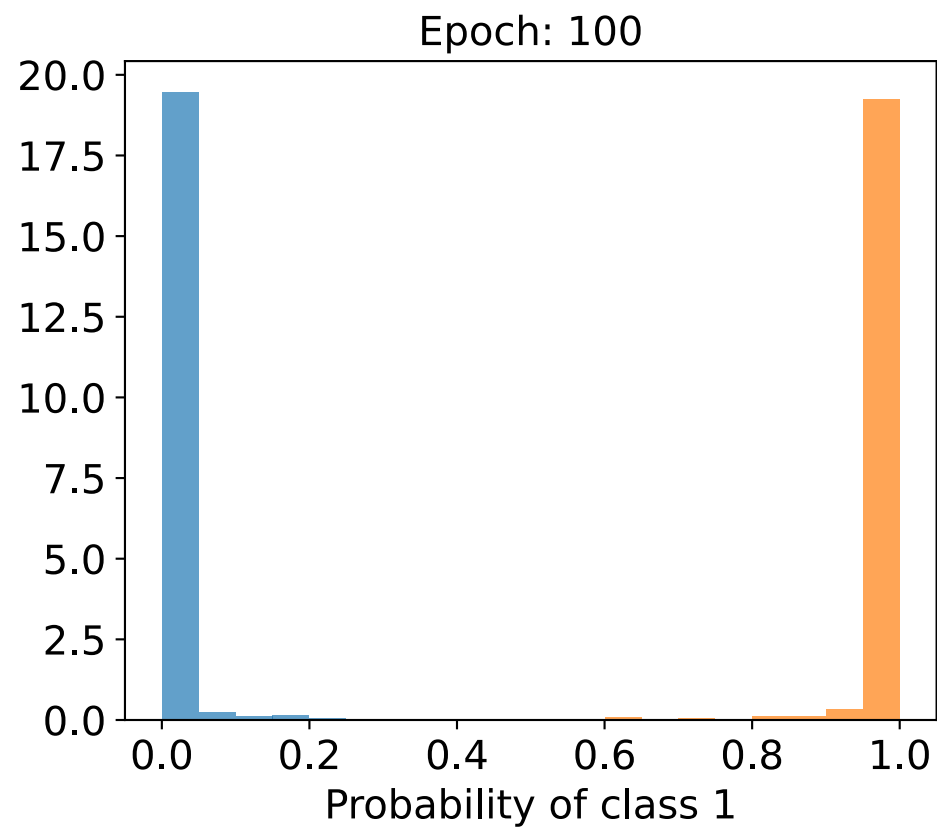

Supplement: S1 Fig — (PDF) [file pone.0289211.s002.pdf]

# ValidationSet

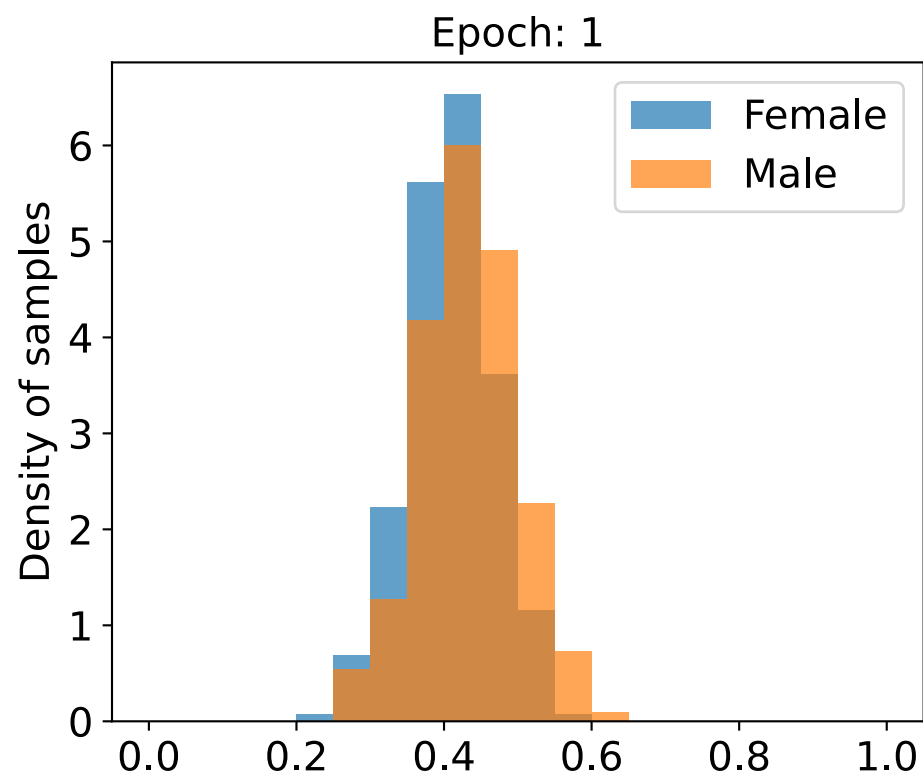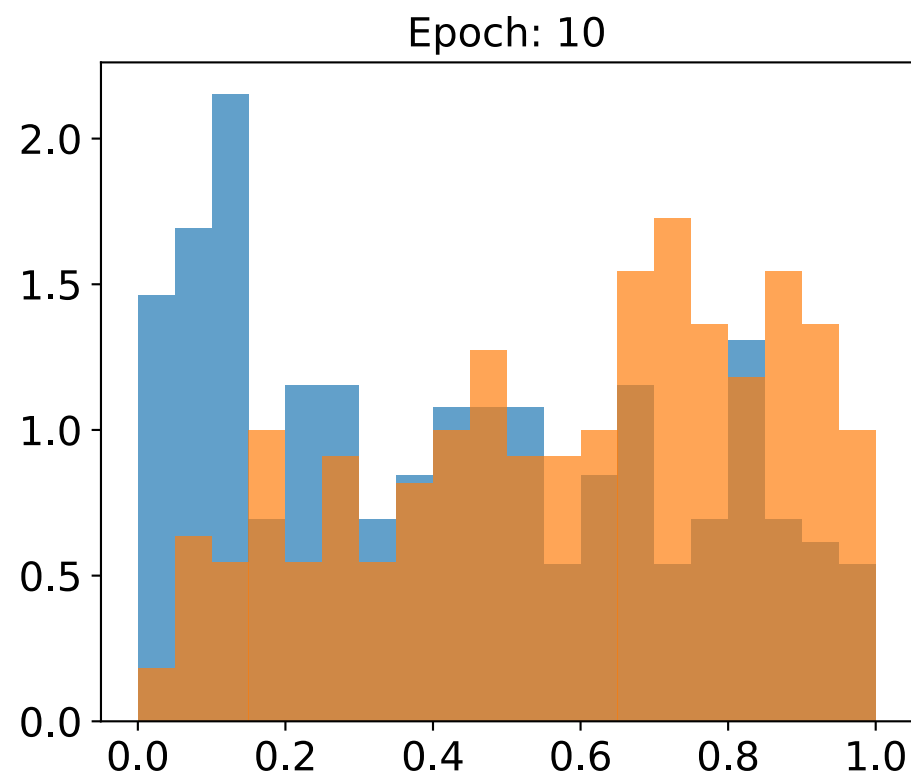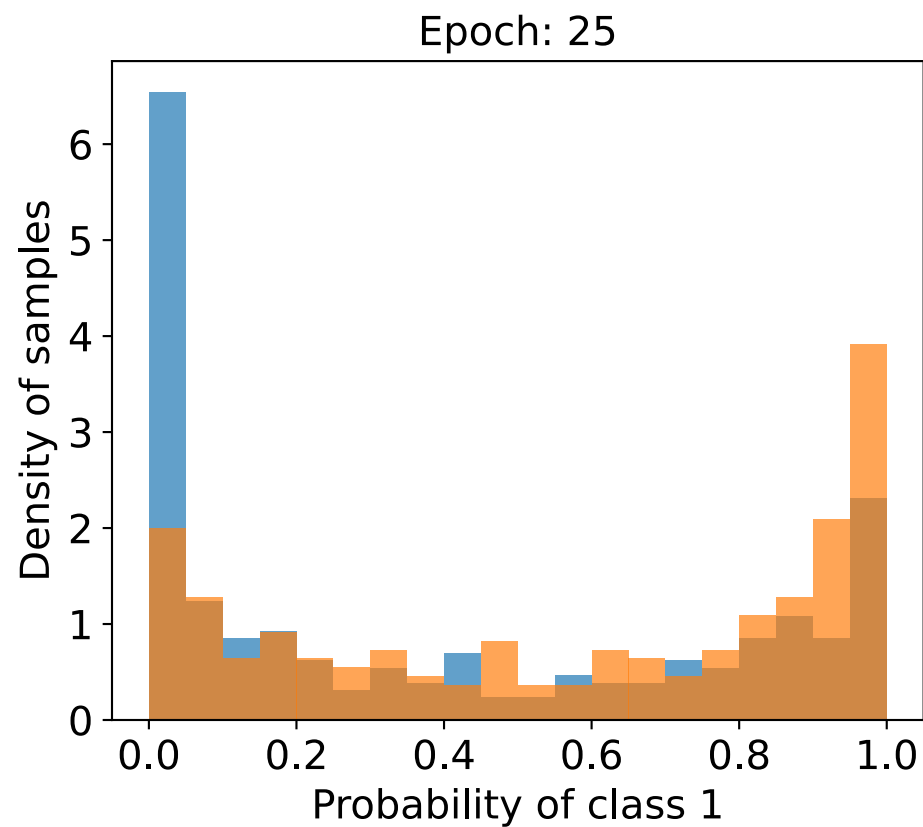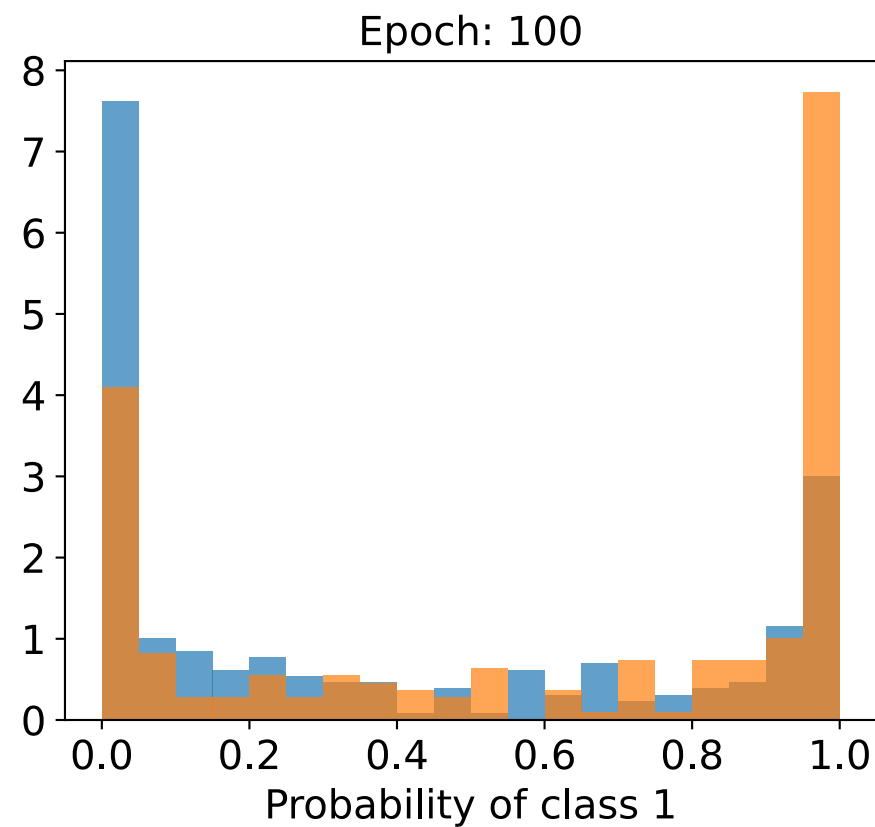

Supplement: S2 Fig — (PDF) [file pone.0289211.s003.pdf]

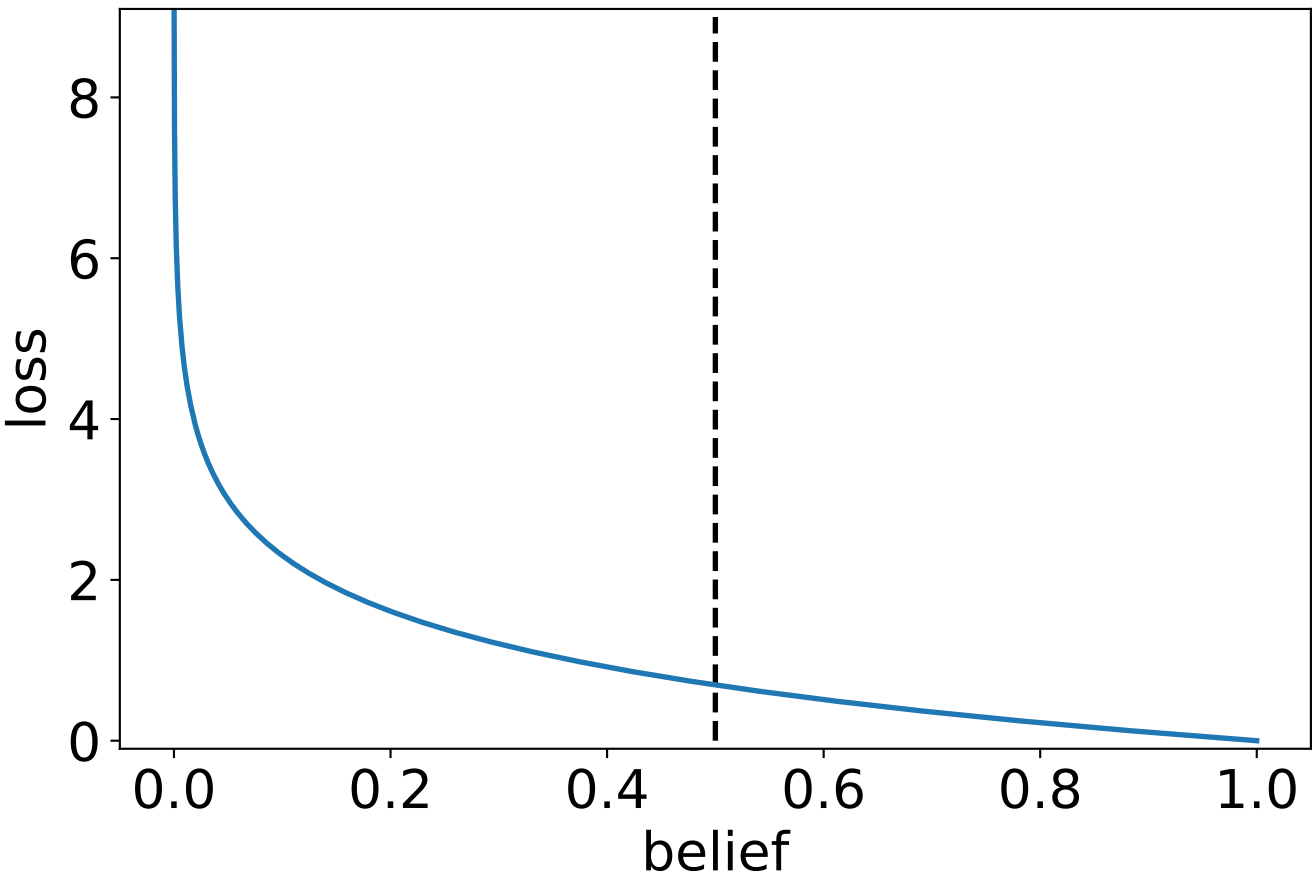

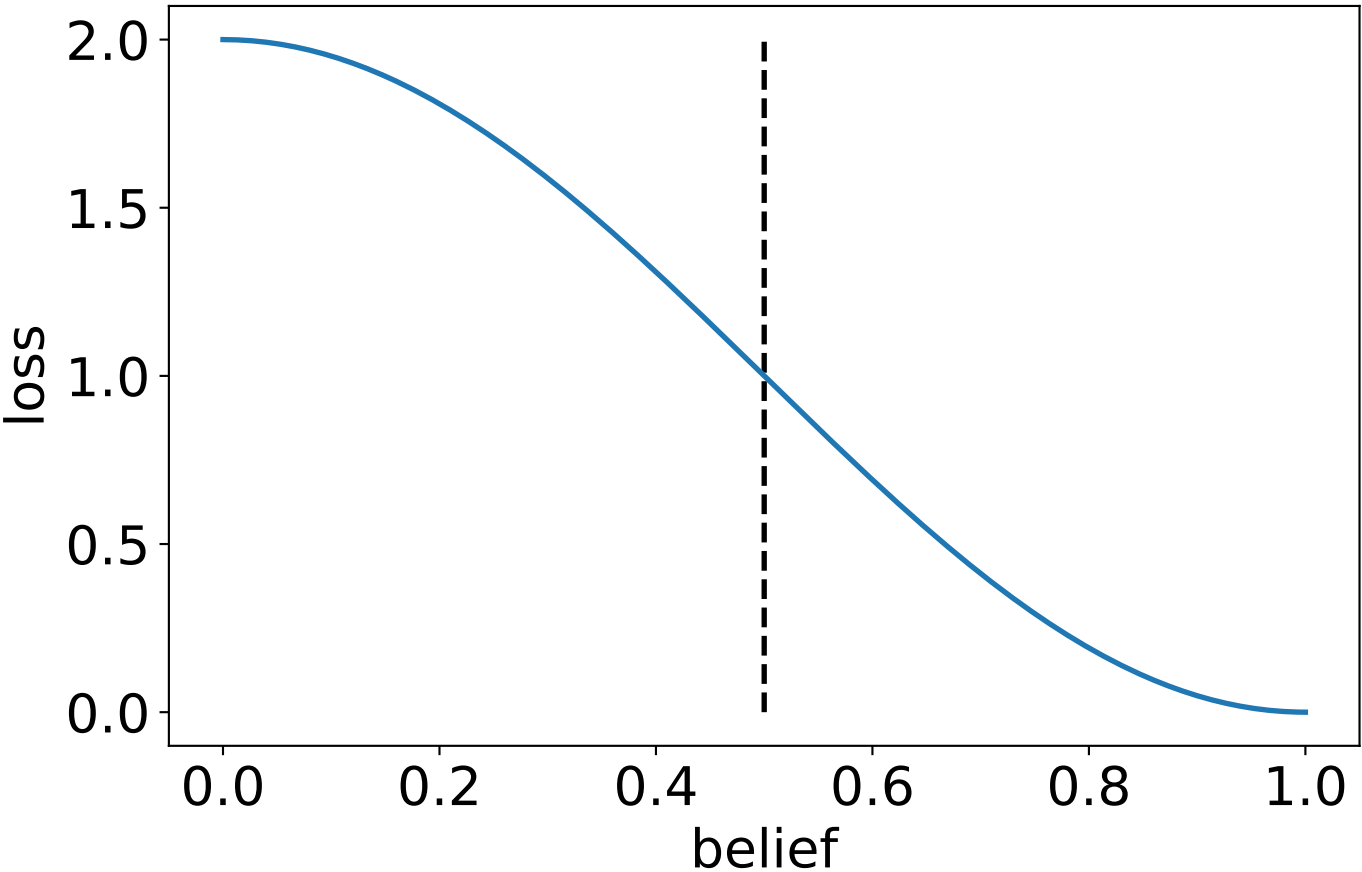

Supplement: S3 Fig — (PDF) [file pone.0289211.s004.pdf]

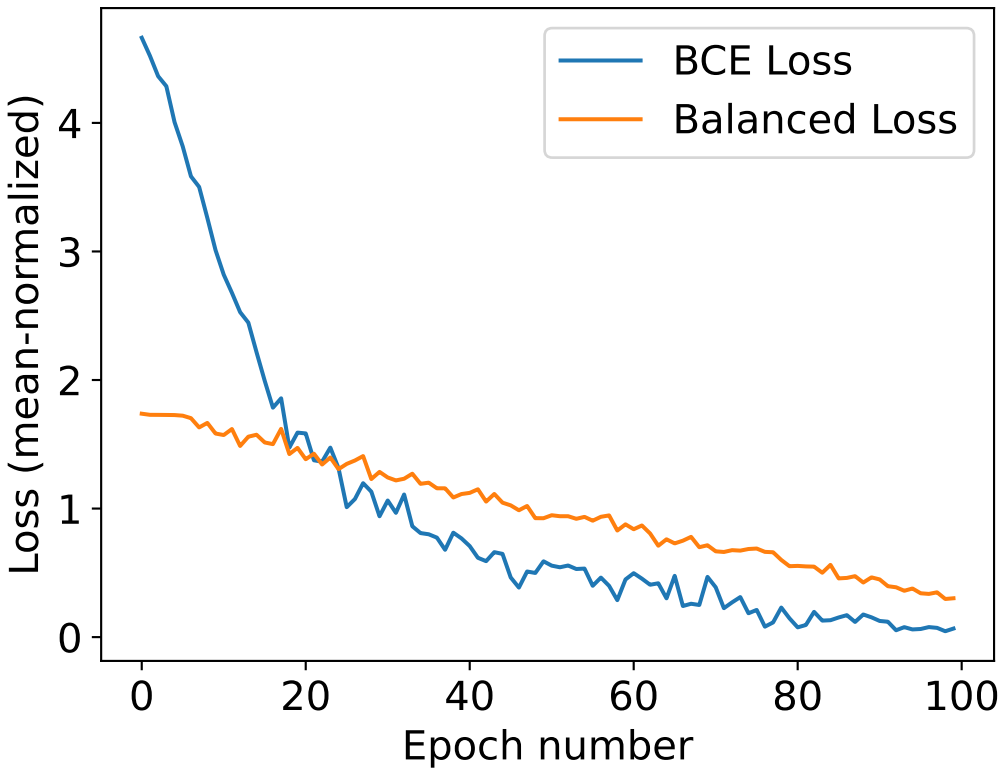

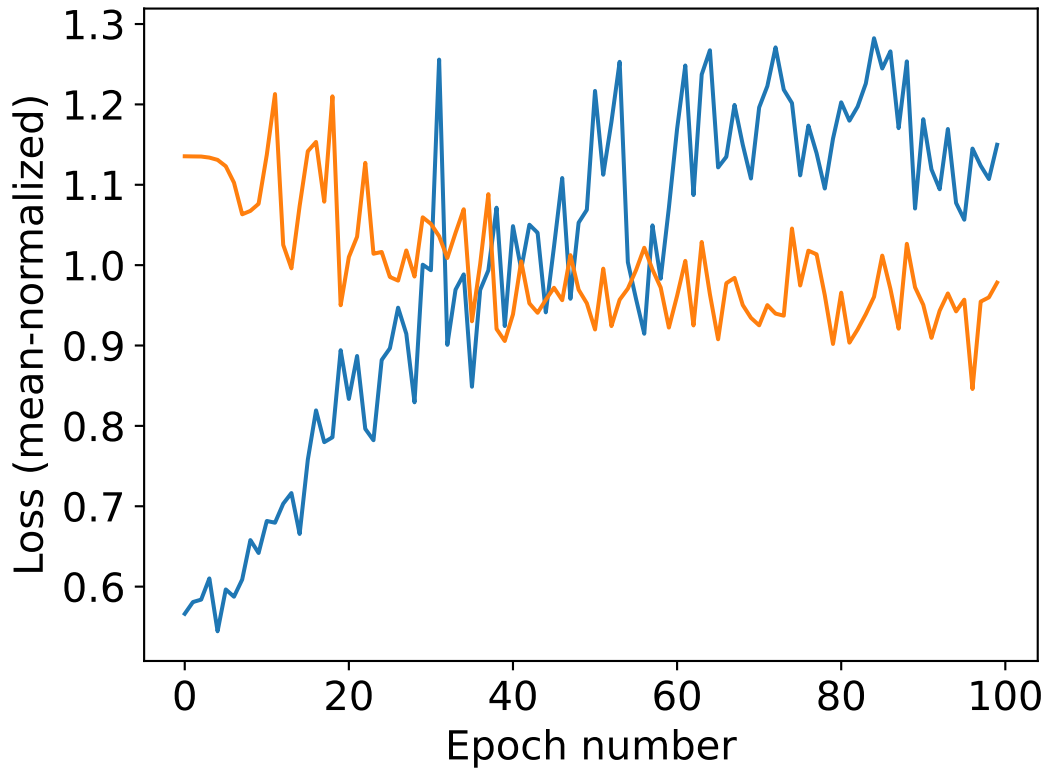

Supplement: S4 Fig — (PDF) [file pone.0289211.s005.pdf]

# Validation Set

Epoch: 1

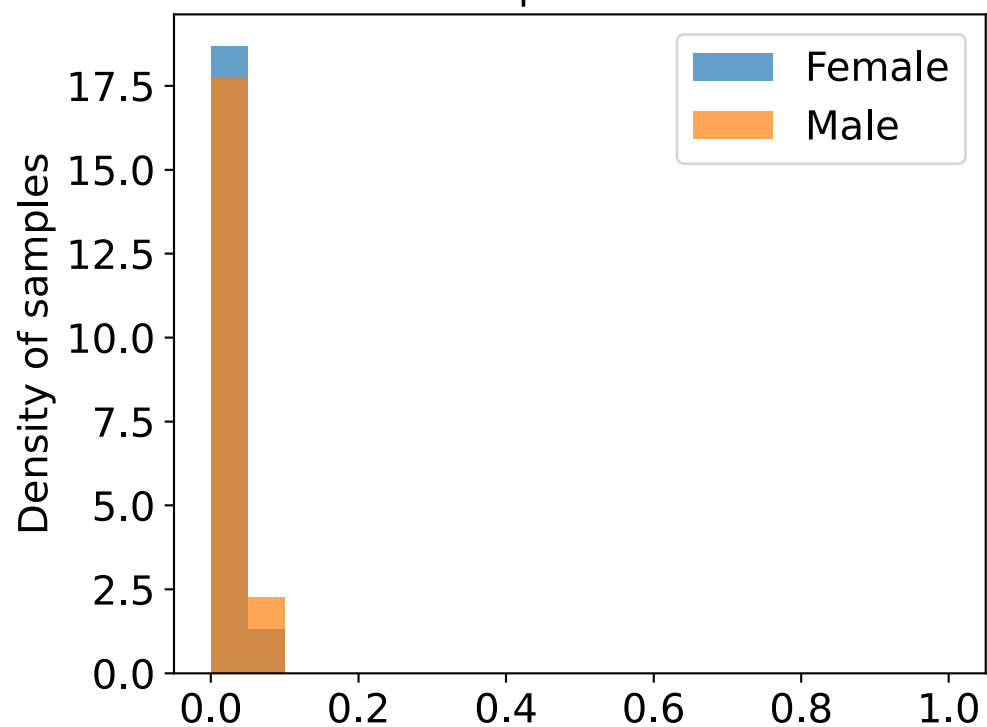

Epoch: 10

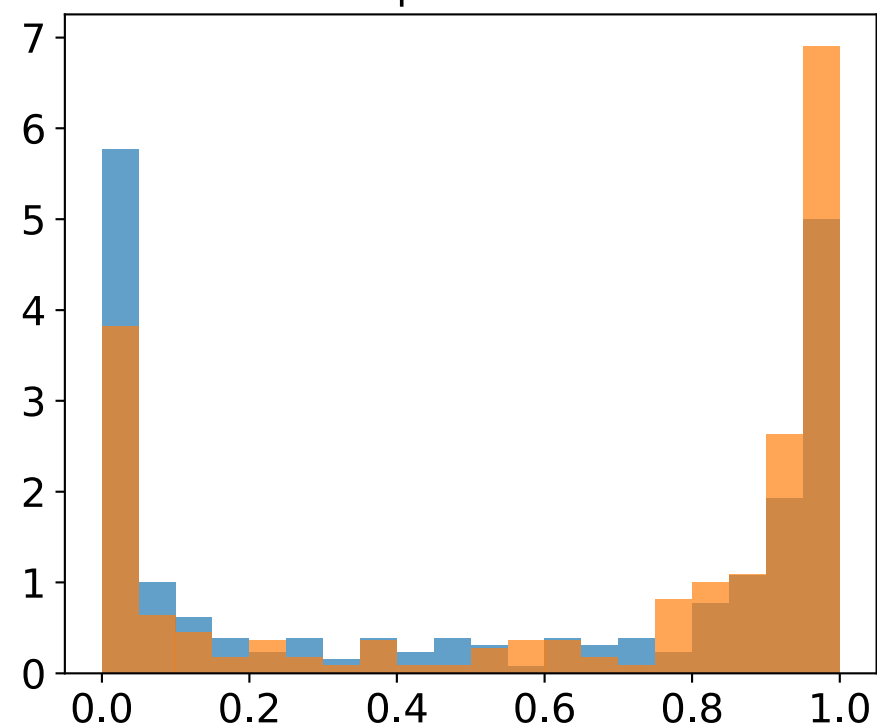

Epoch: 25

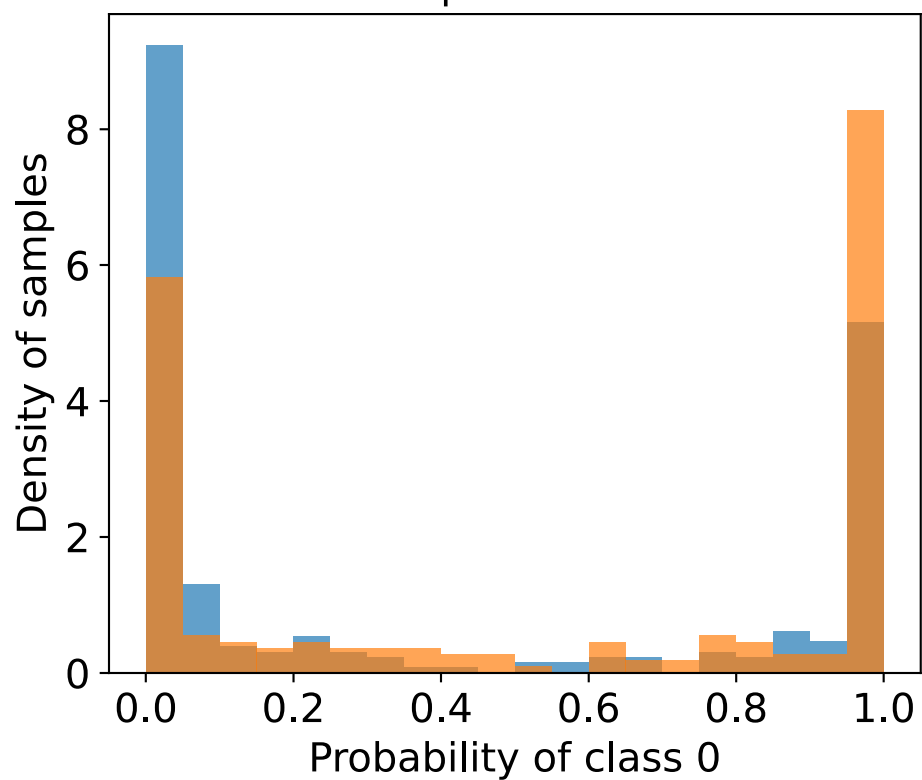

Epoch: 100

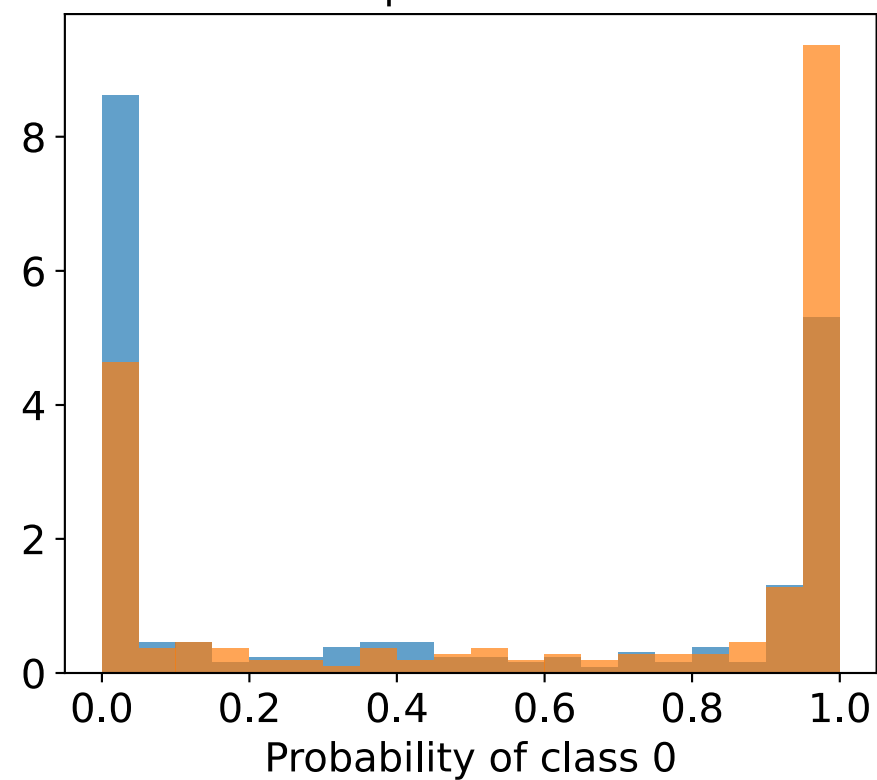

Supplement: S5 Fig — (PDF) [file pone.0289211.s006.pdf]

train

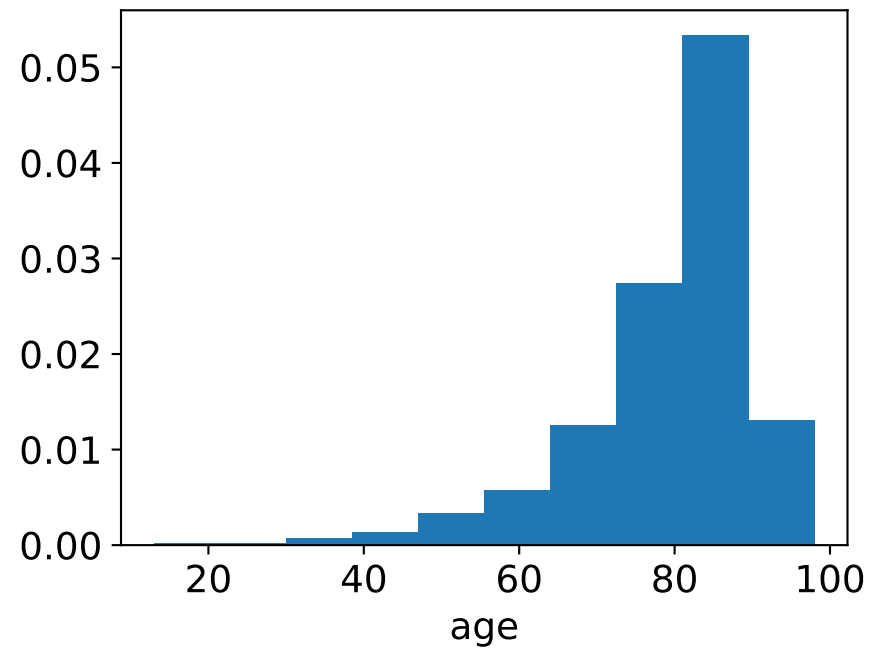

val

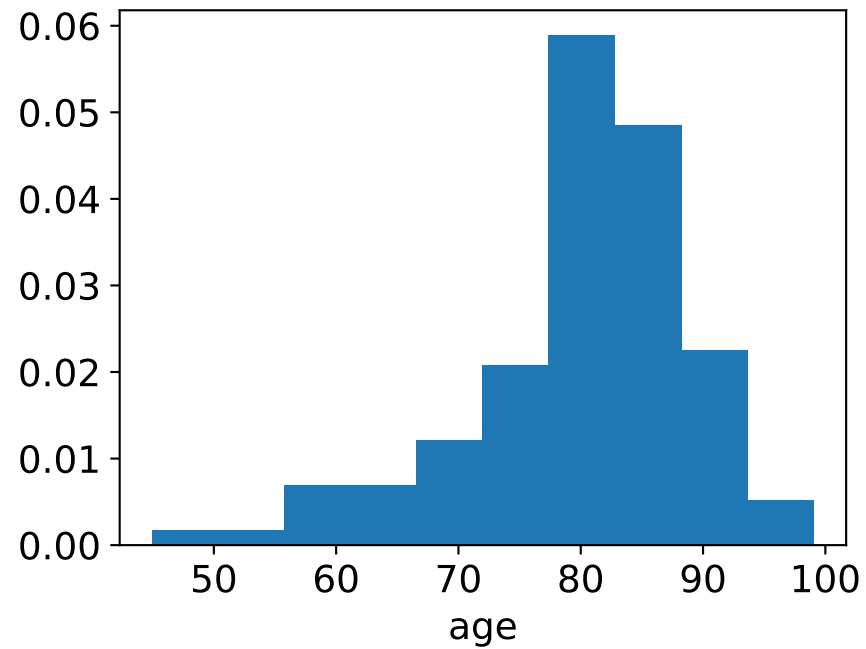

test

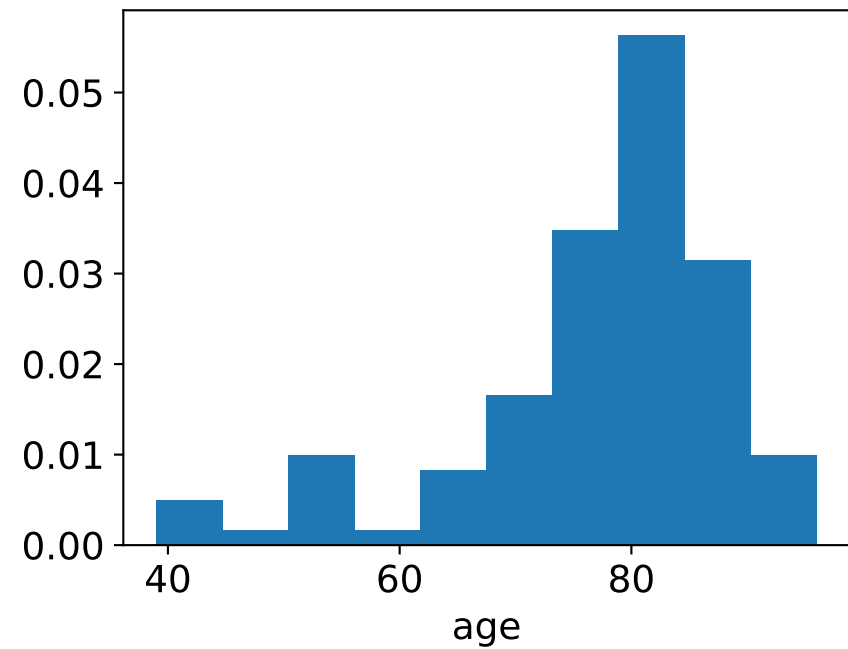

train

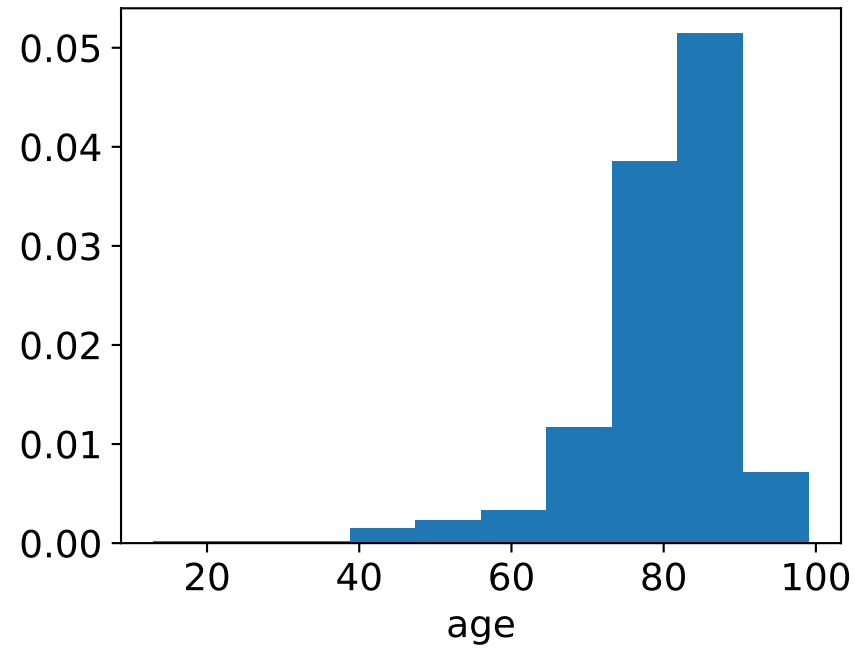

val

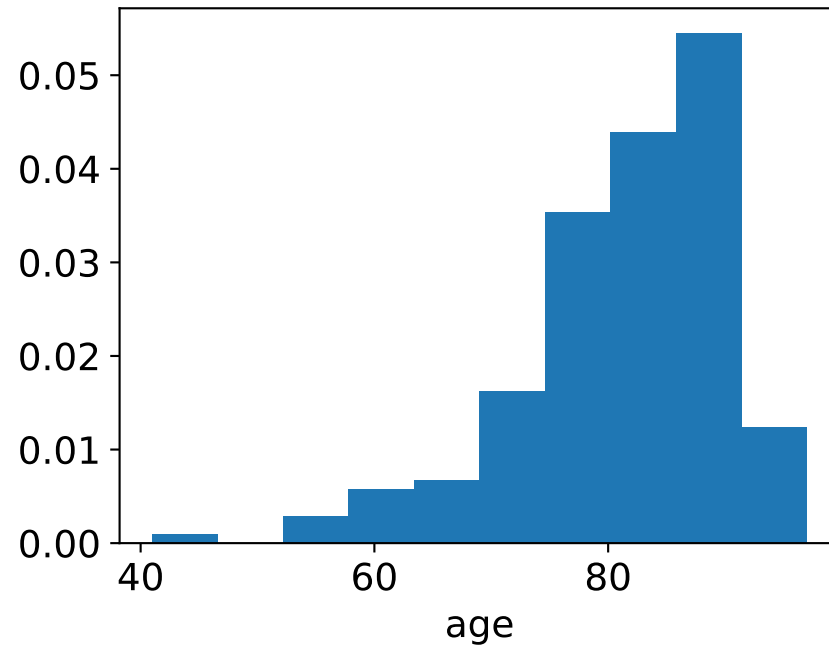

test

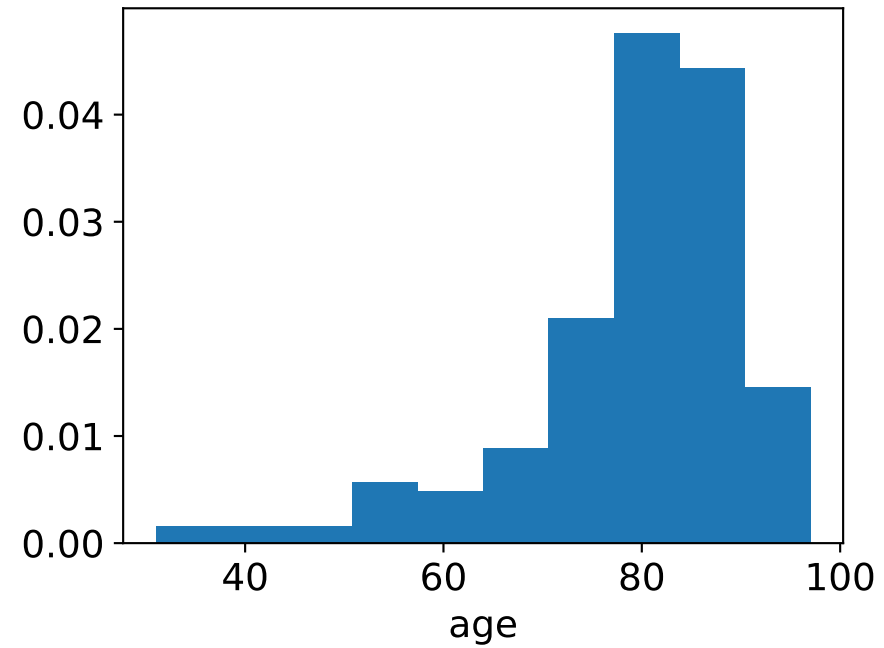

Supplement: S6 Fig — (PDF) [file pone.0289211.s007.pdf]

Predicted: F

Predicted: F

Predicted: F

Predicted: M

Predicted: F

Predicted: M

F

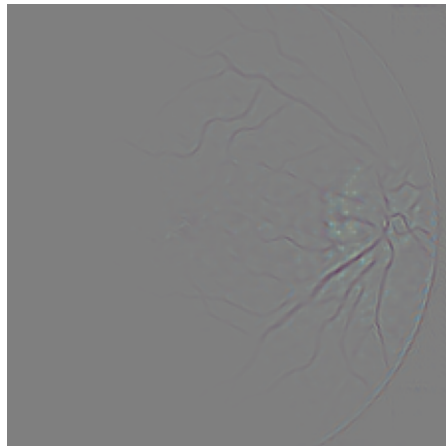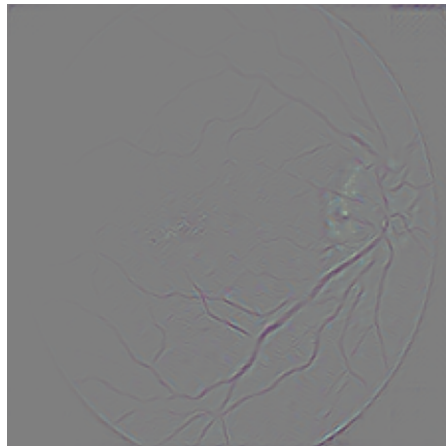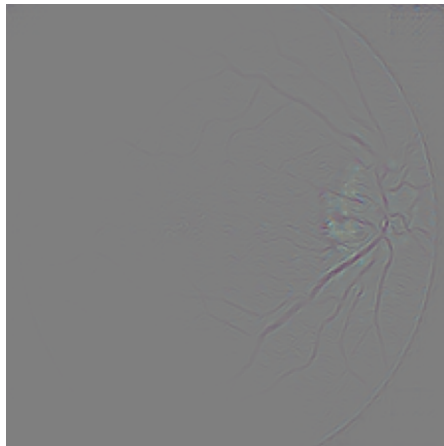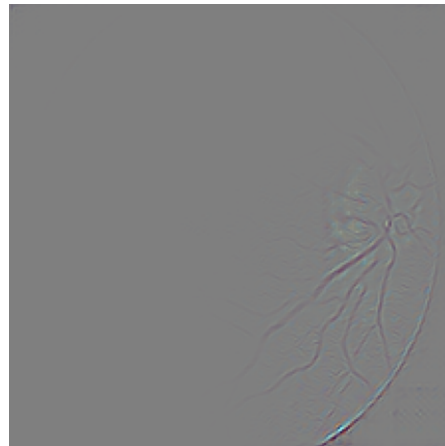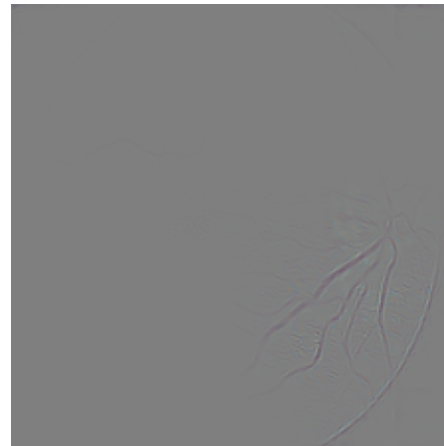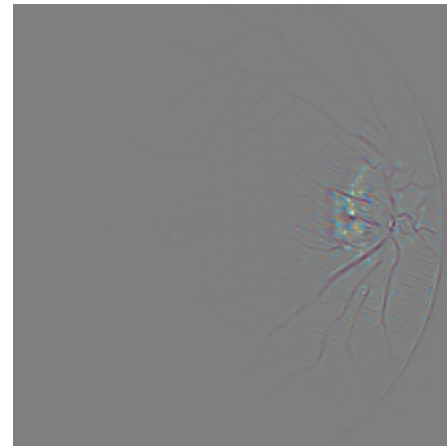

M

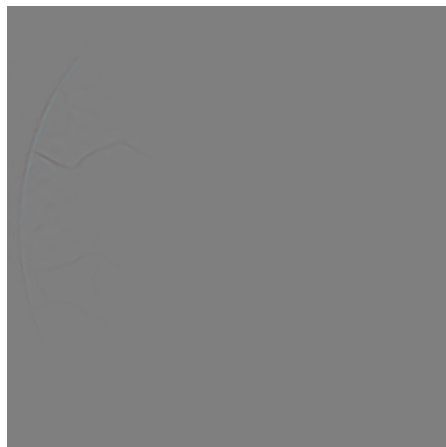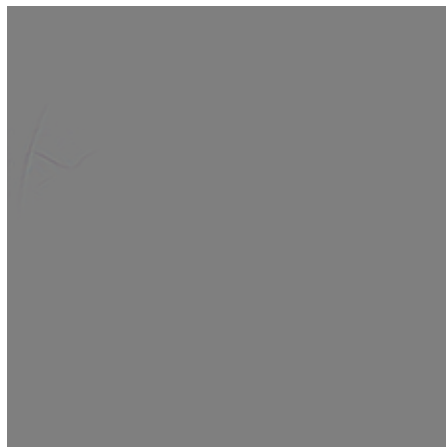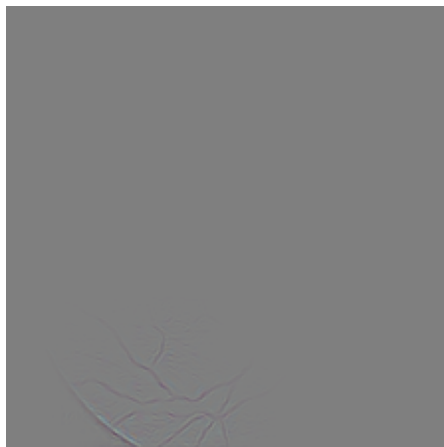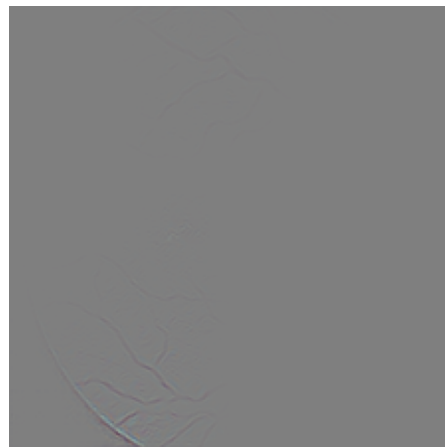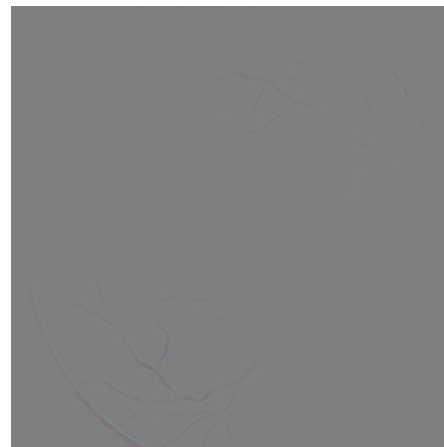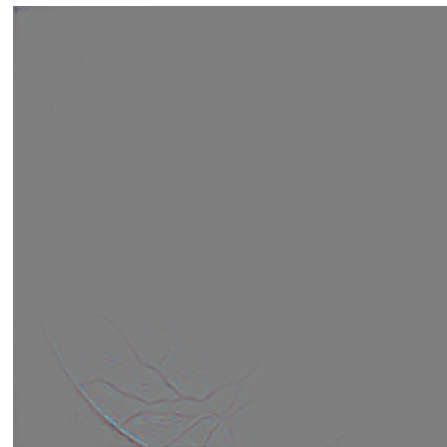

Supplement: S7 Fig — (ZIP) [file pone.0289211.s008.zip › figS7ai.pdf]

Predicted: F

Predicted: F

Predicted: F

Predicted: M

Predicted: F

Predicted: M

F

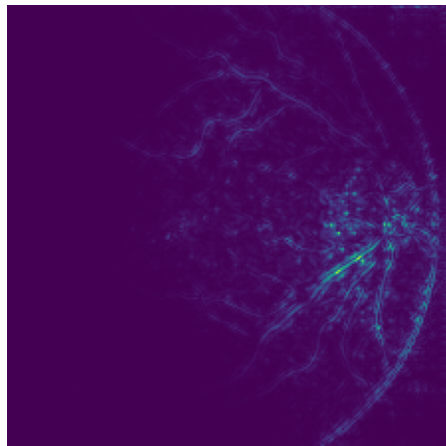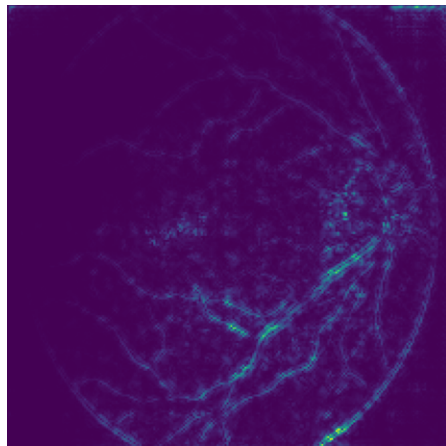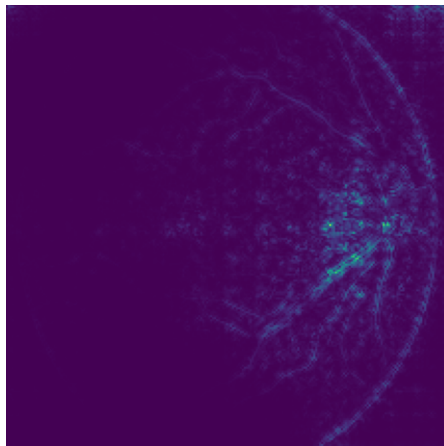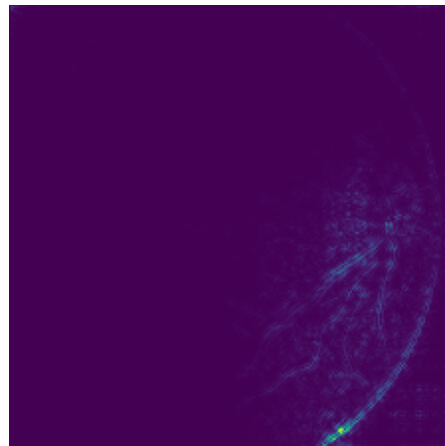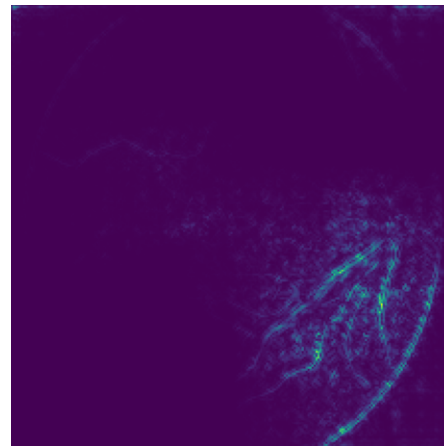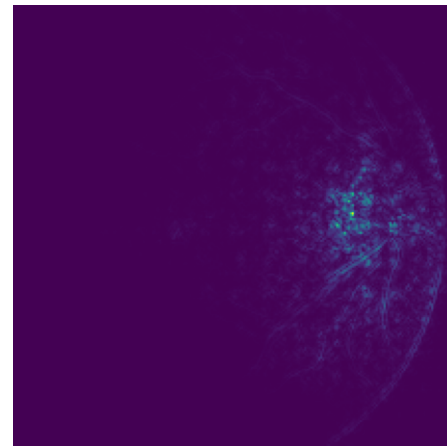

M

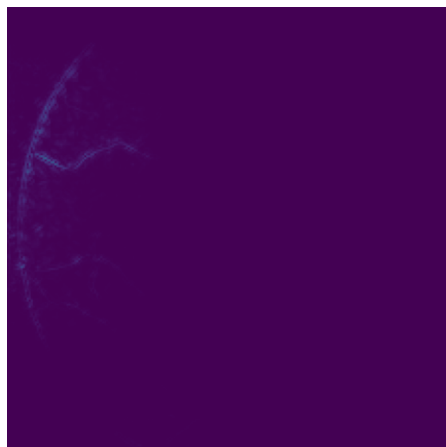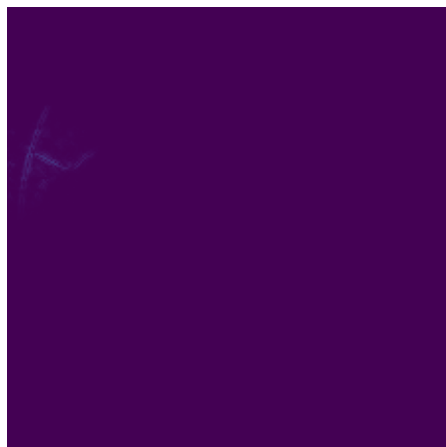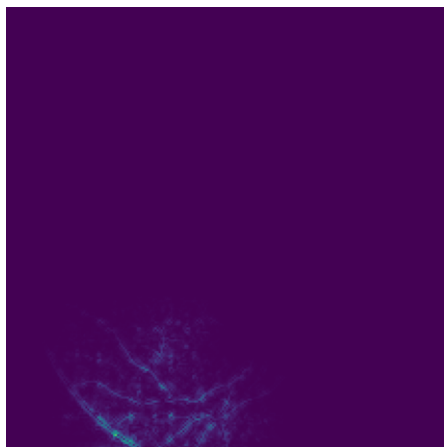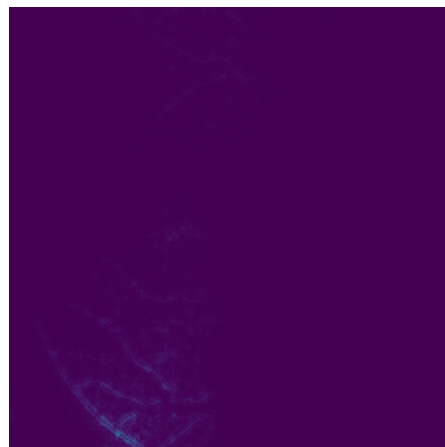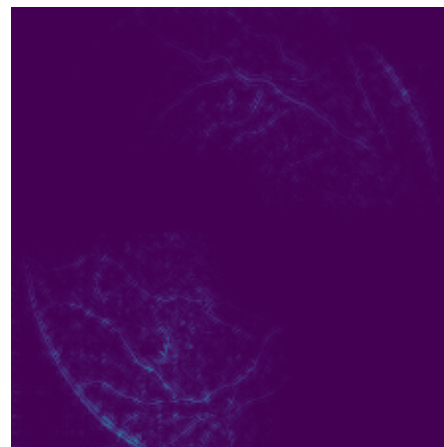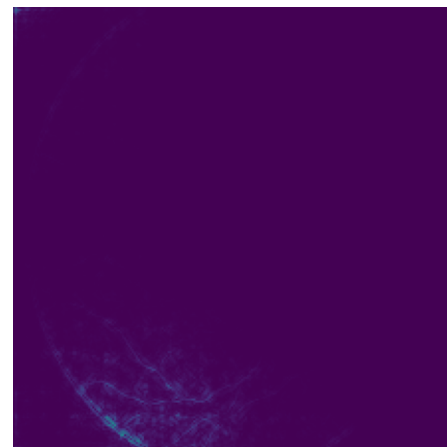

Supplement: S7 Fig — (ZIP) [file pone.0289211.s008.zip › figS7aii.pdf]

Predicted: F

Predicted: F

Predicted: F

Predicted: M

Predicted: F

Predicted: M

F

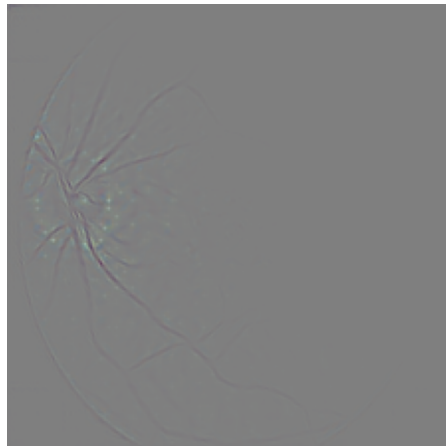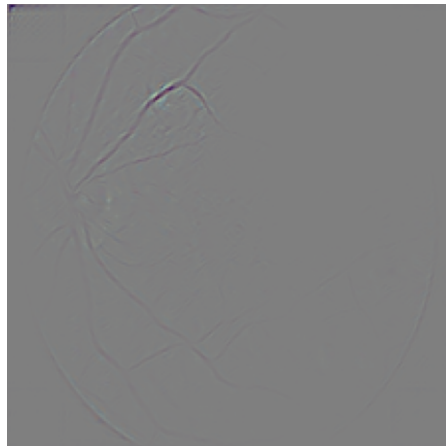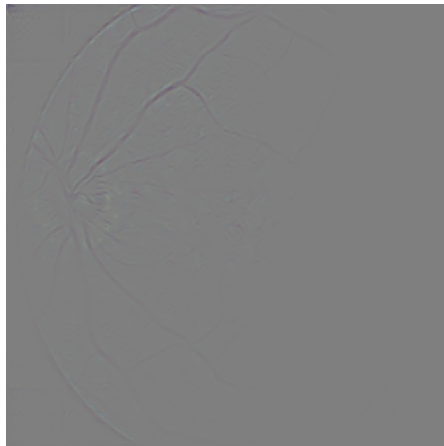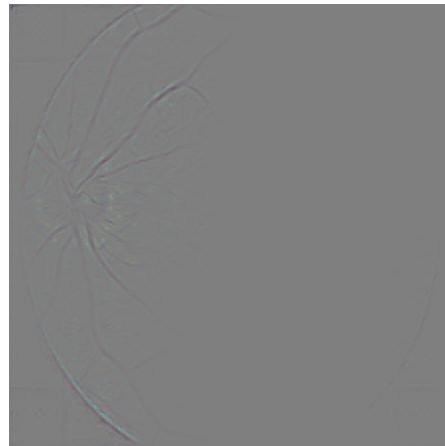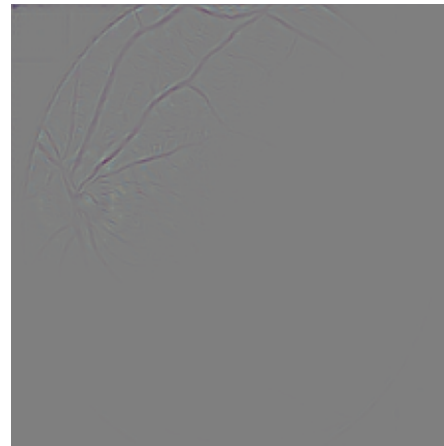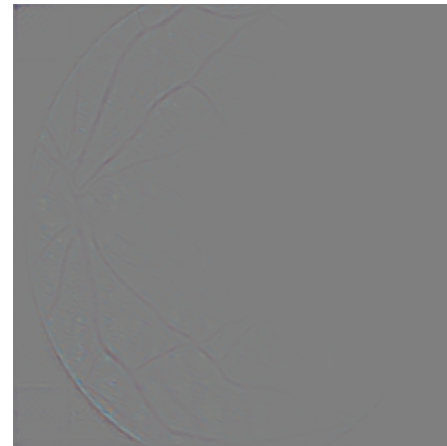

M

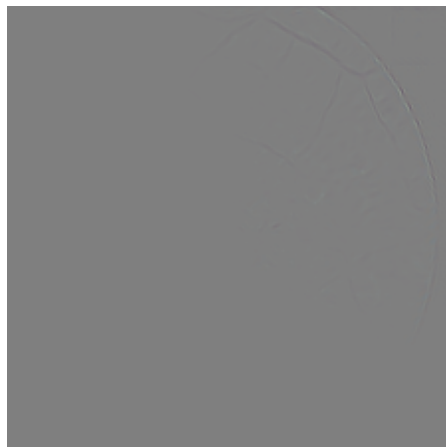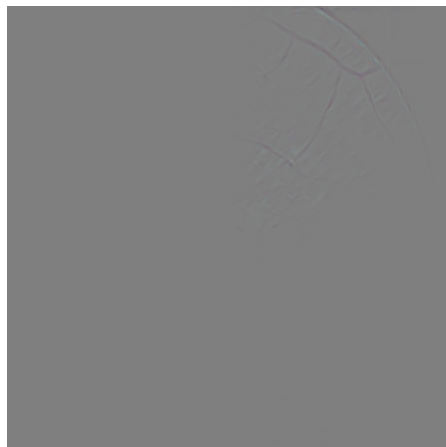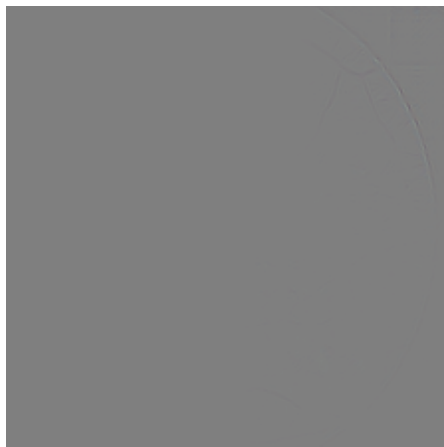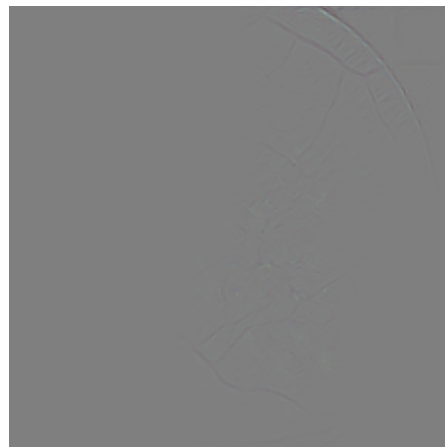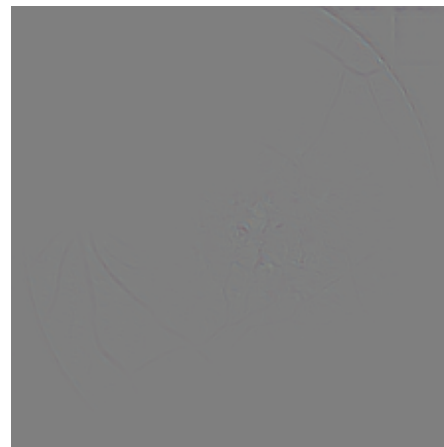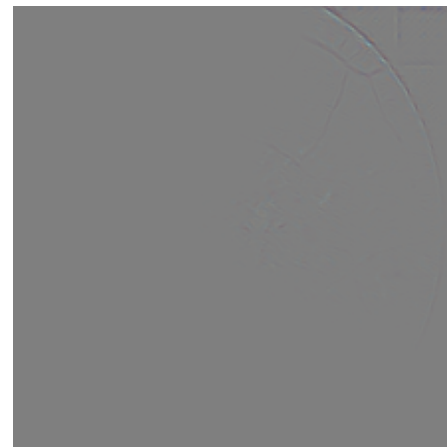

Supplement: S7 Fig — (ZIP) [file pone.0289211.s008.zip › figS7bi.pdf]

Predicted: F

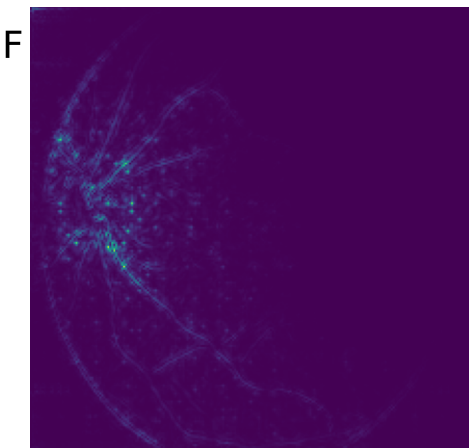

Predicted: F

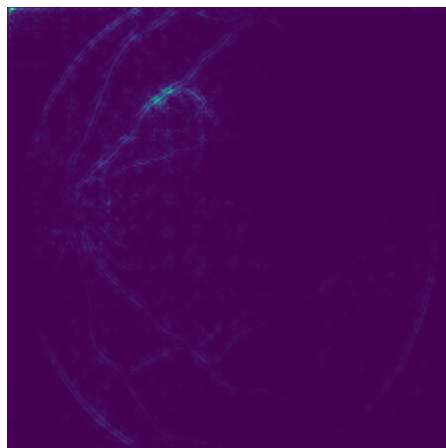

Predicted: F

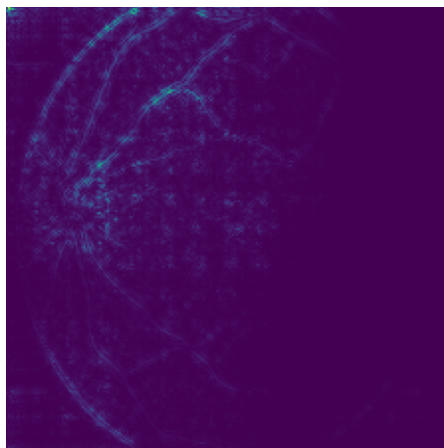

Predicted: M

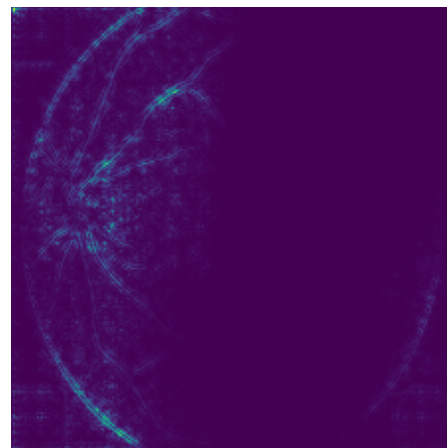

Predicted: F

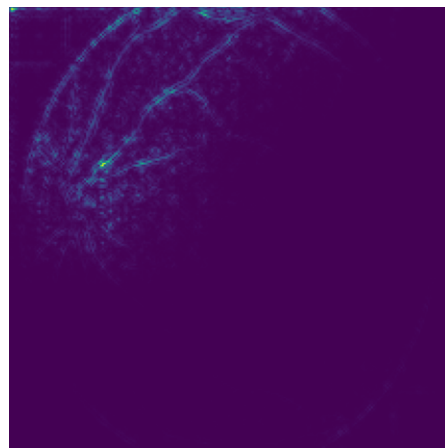

Predicted: M

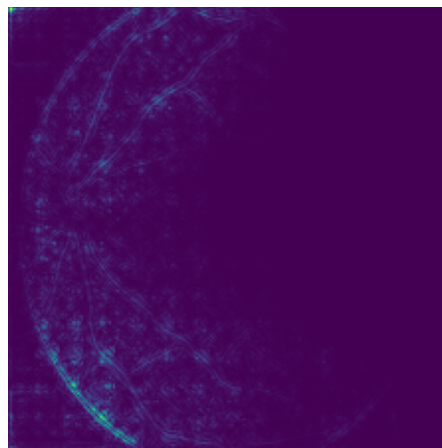

M

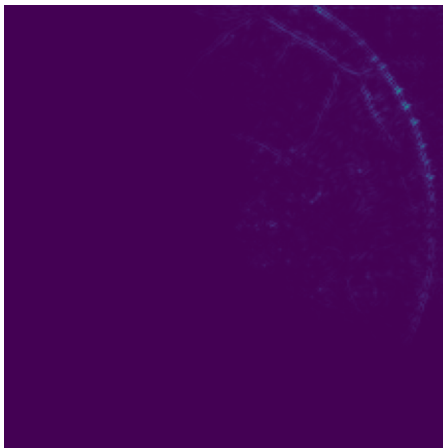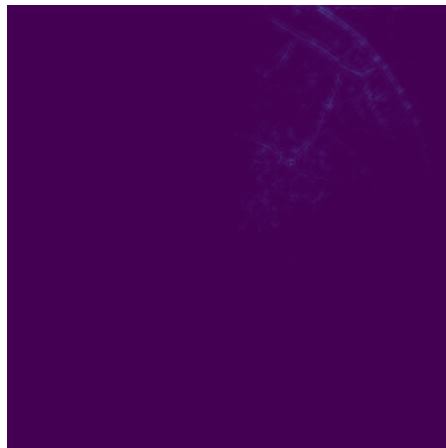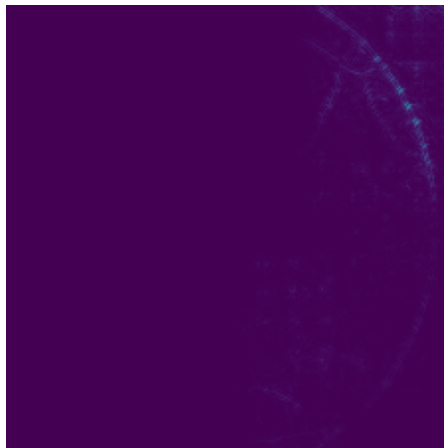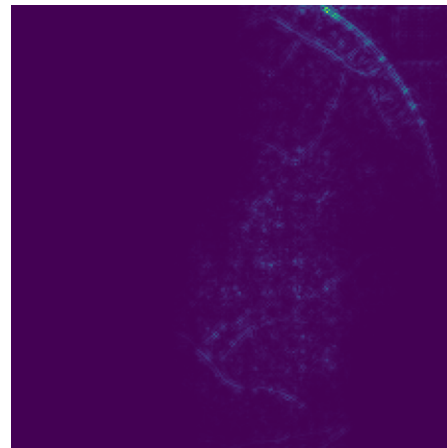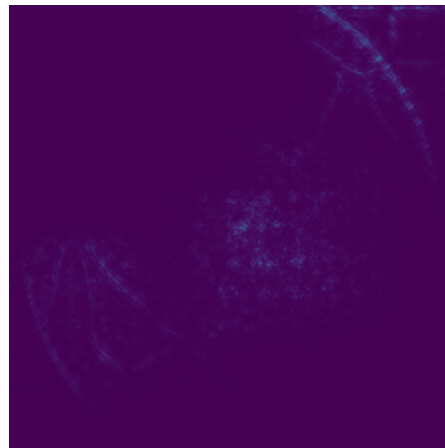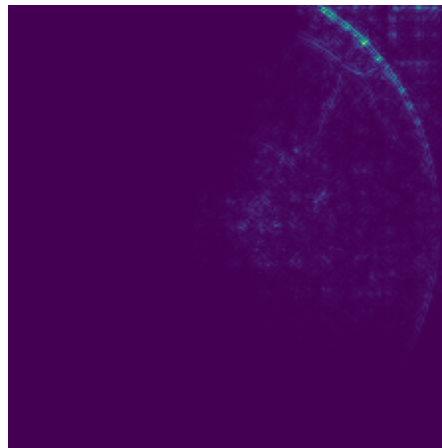

Supplement: S7 Fig — (ZIP) [file pone.0289211.s008.zip › figS7bii.pdf]

Predicted: M

Predicted: F

Predicted: M

Predicted: M

Predicted: F

Predicted: F

F

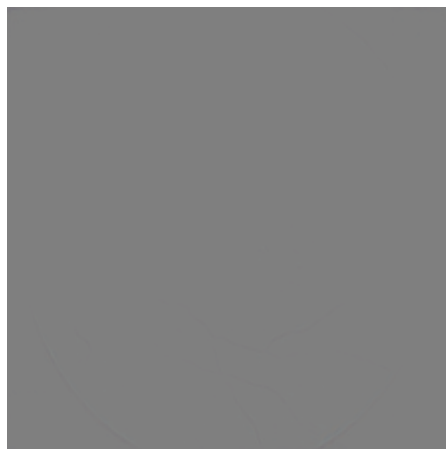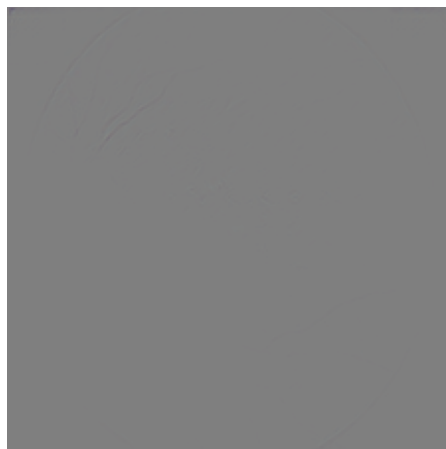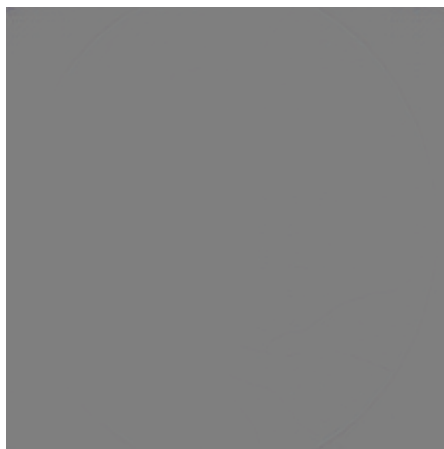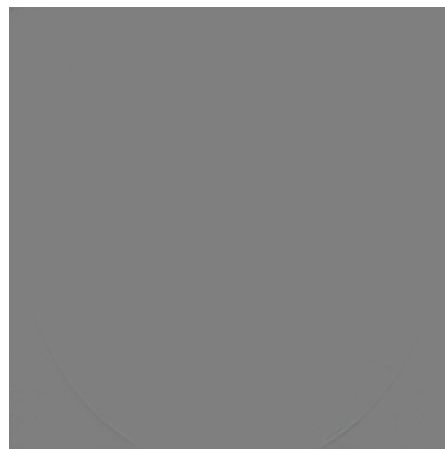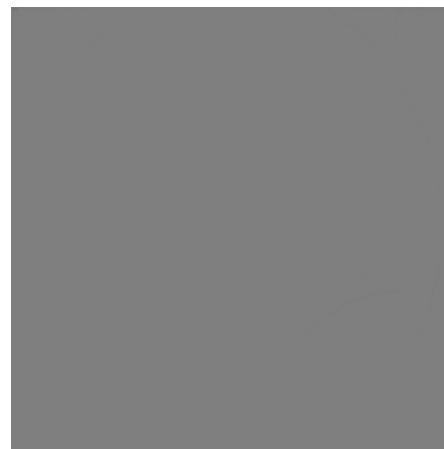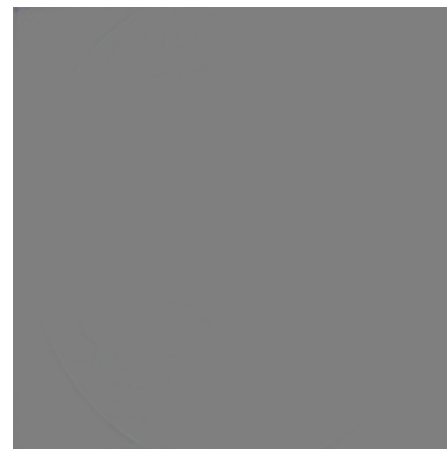

M

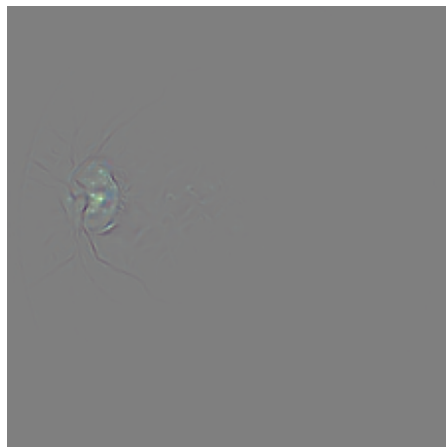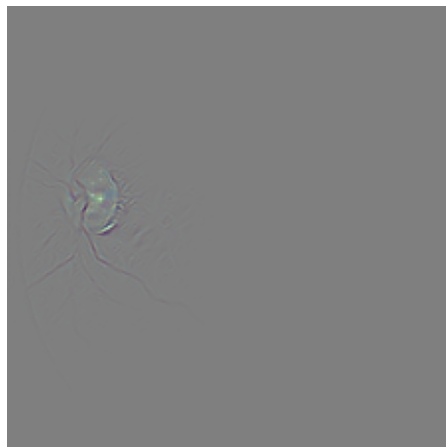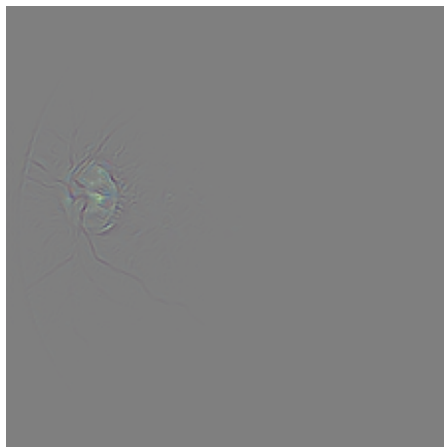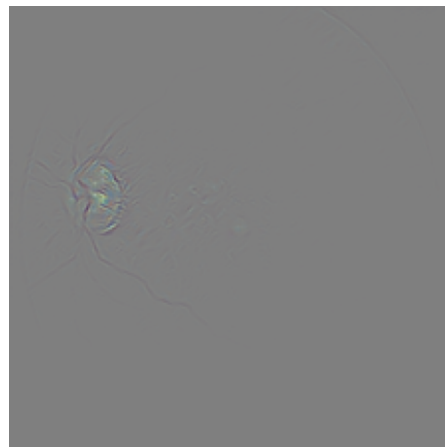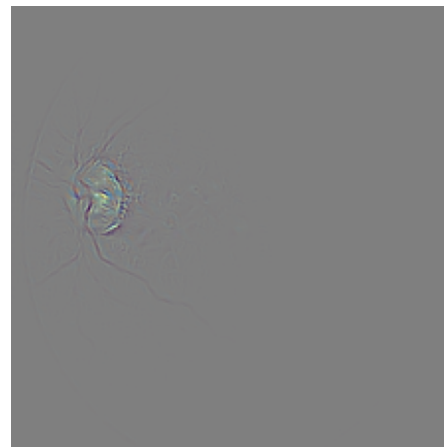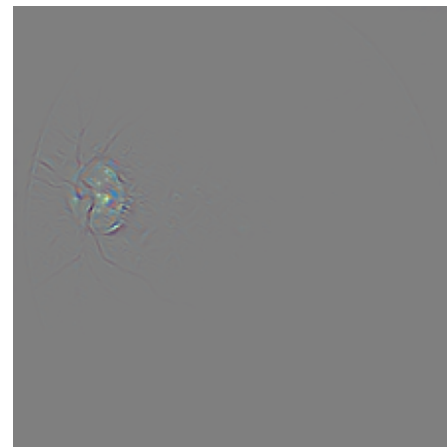

Supplement: S8 Fig — (ZIP) [file pone.0289211.s009.zip › figS8ai.pdf]

Predicted: M

Predicted: F

Predicted: M

Predicted: M

Predicted: F

Predicted: F

F

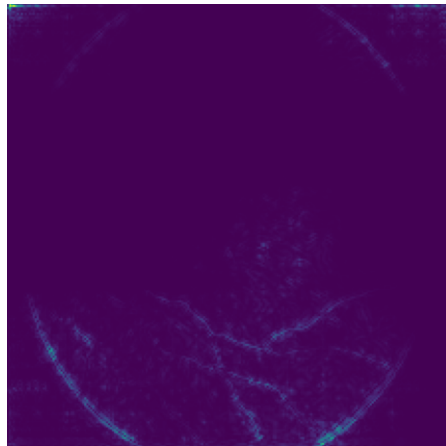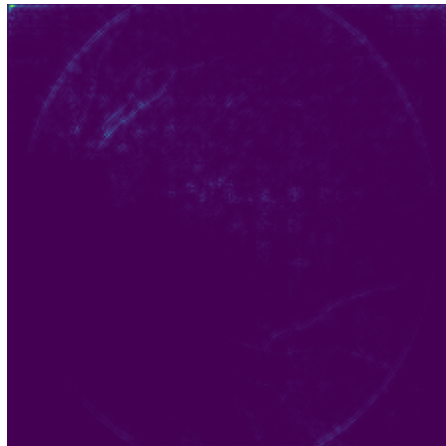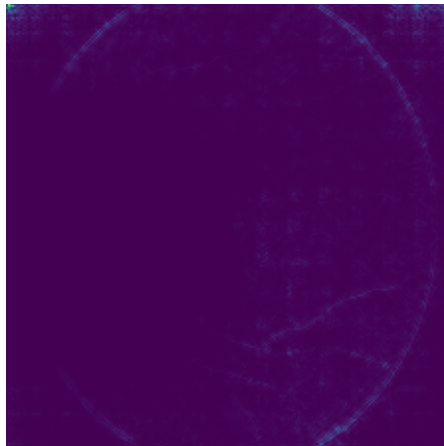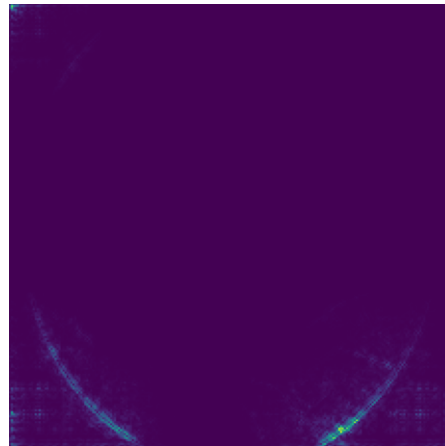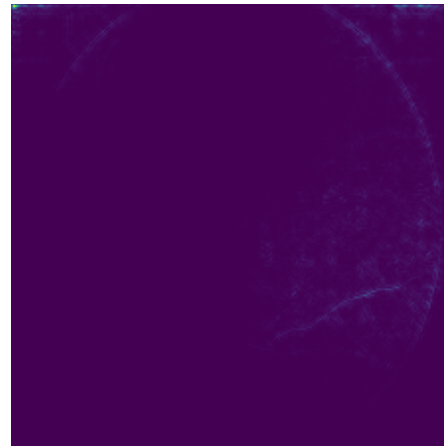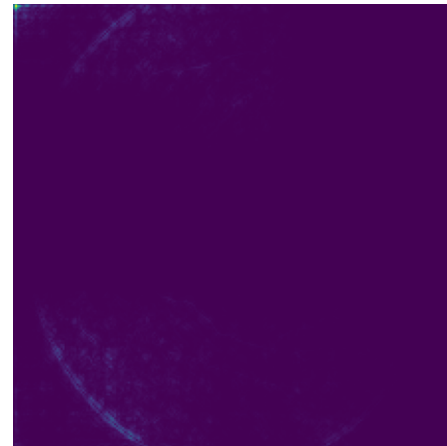

M

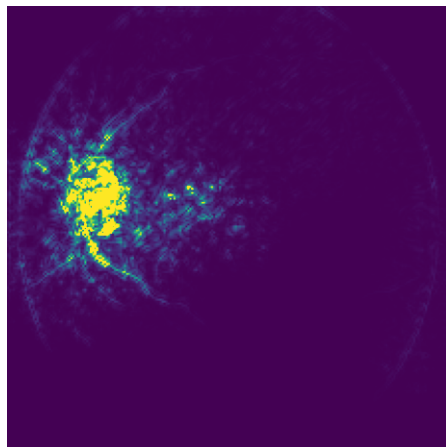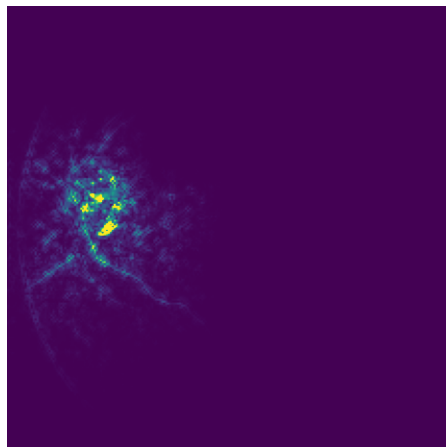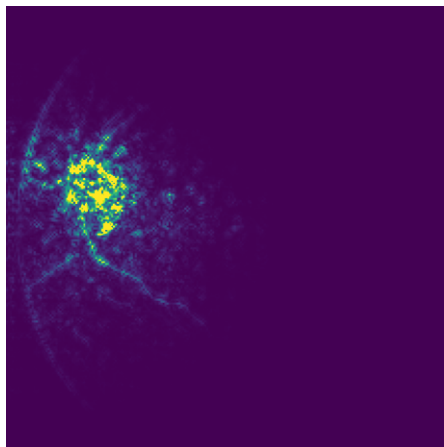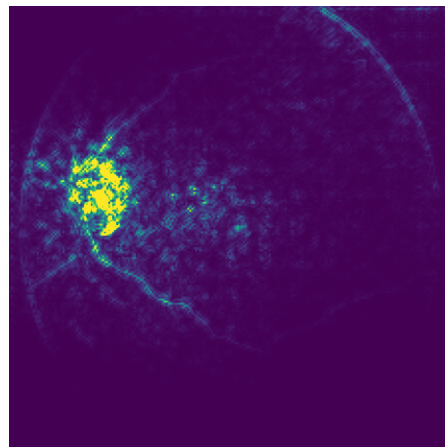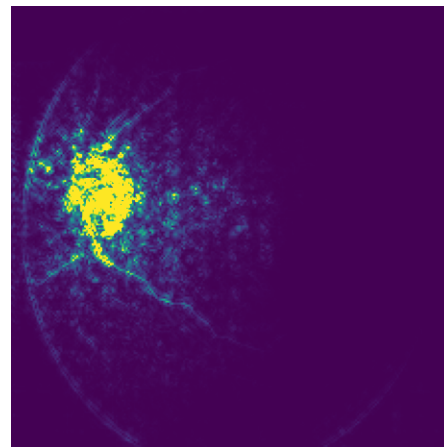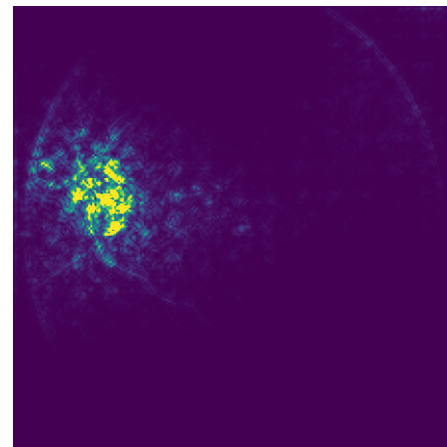

Supplement: S8 Fig — (ZIP) [file pone.0289211.s009.zip › figS8aii.pdf]

Predicted: M

Predicted: M

Predicted: M

Predicted: M

Predicted: F

Predicted: M

F

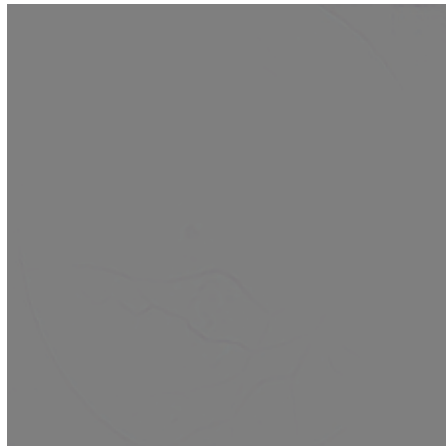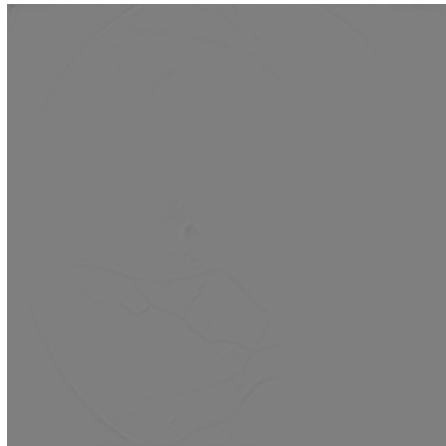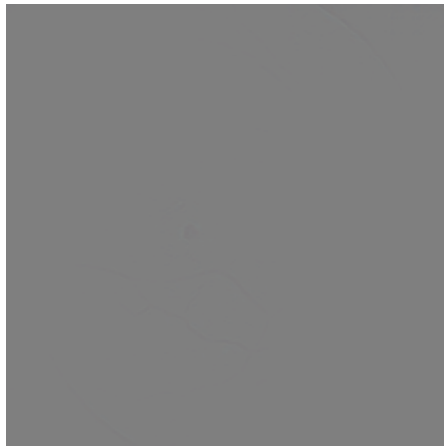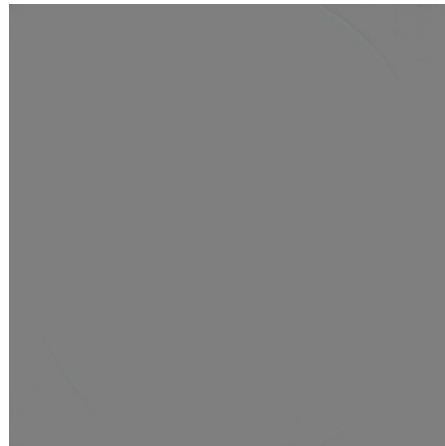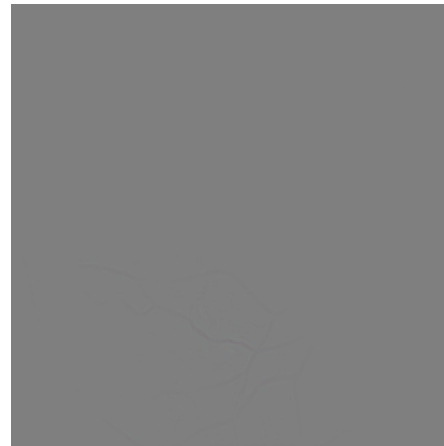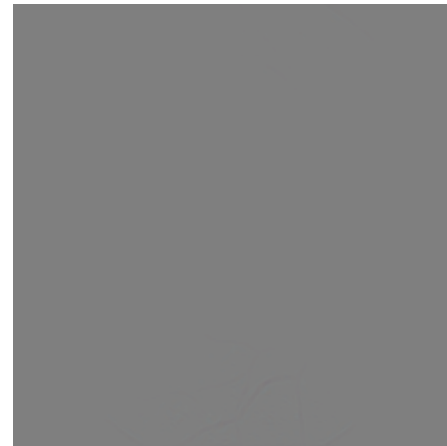

M

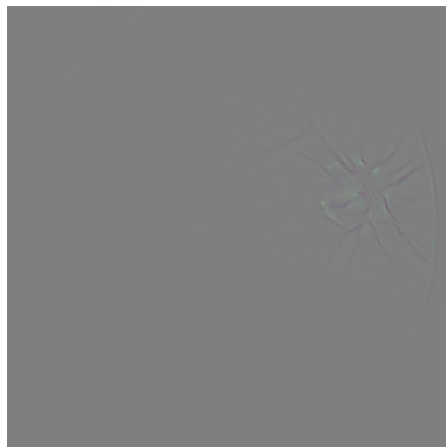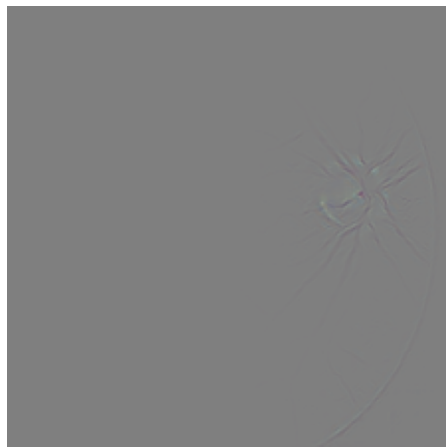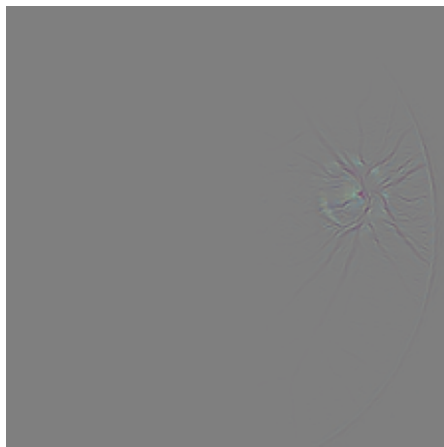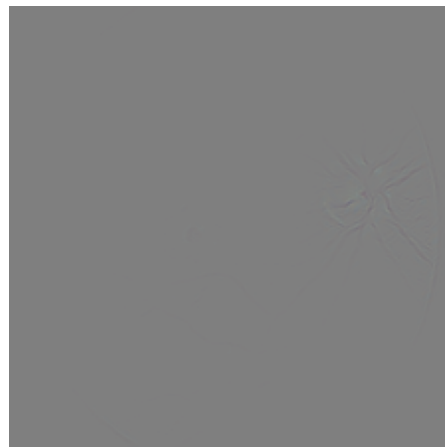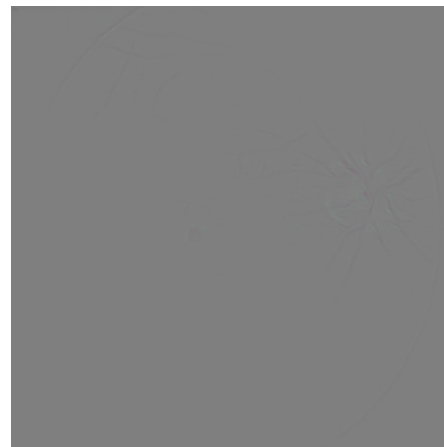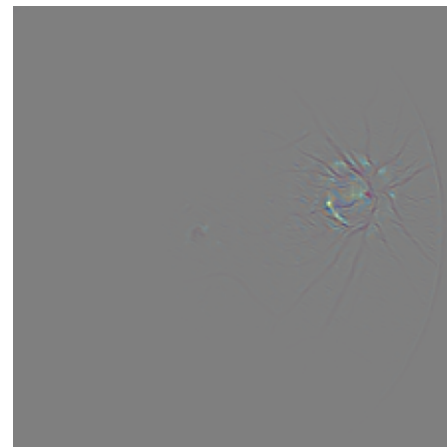

Supplement: S8 Fig — (ZIP) [file pone.0289211.s009.zip › figS8bi.pdf]

Predicted: M

Predicted: M

Predicted: M

Predicted: M

Predicted: F

Predicted: M

F

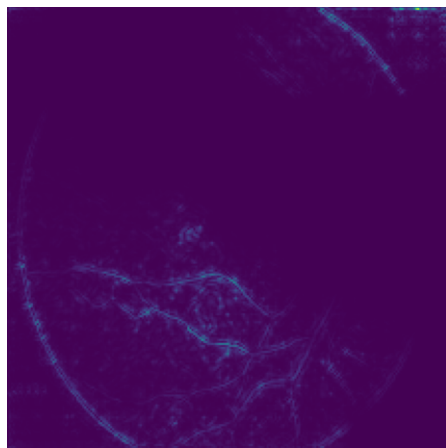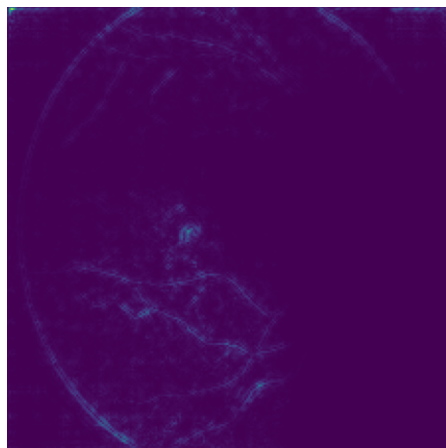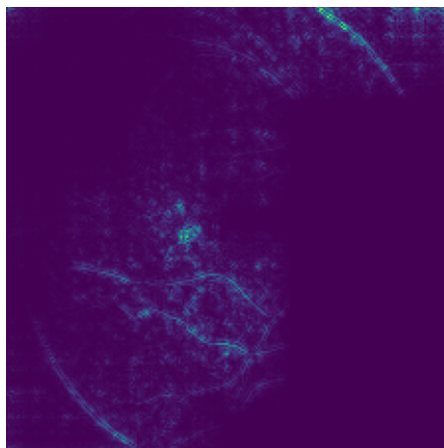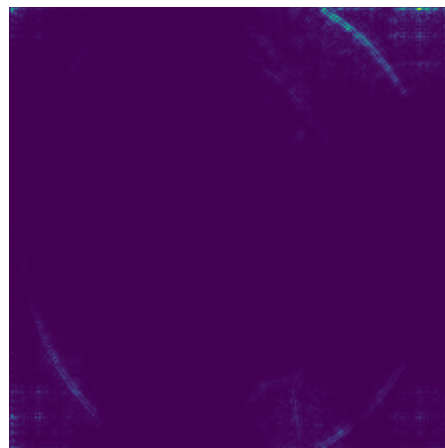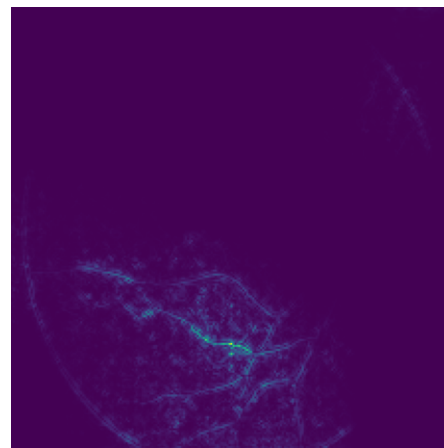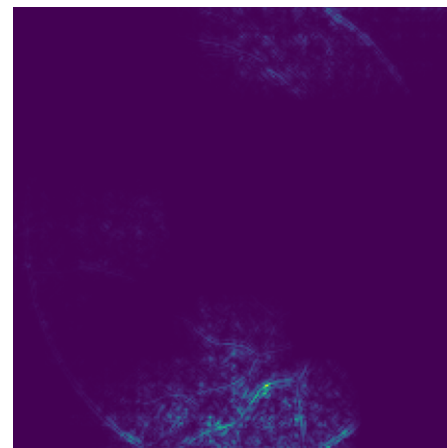

M

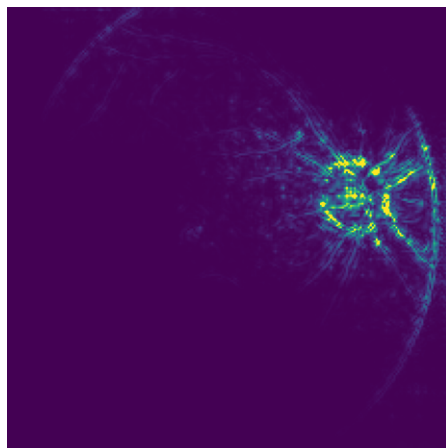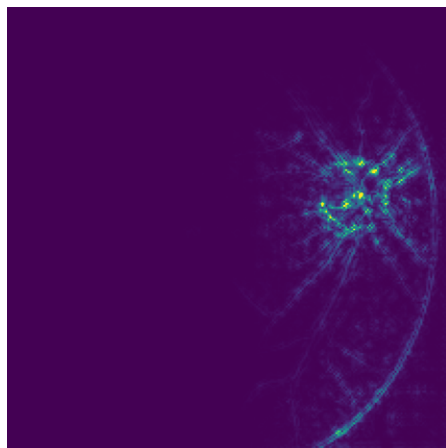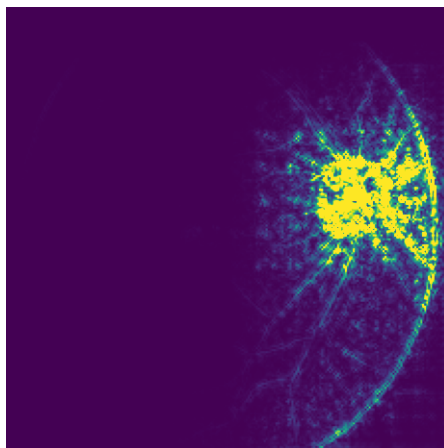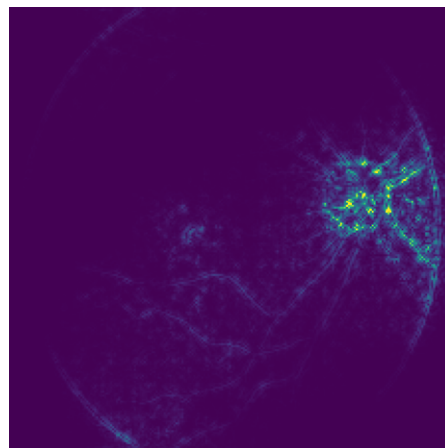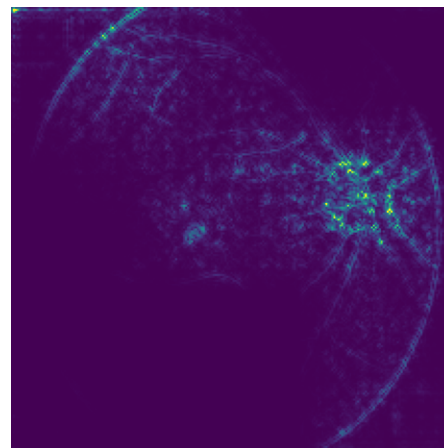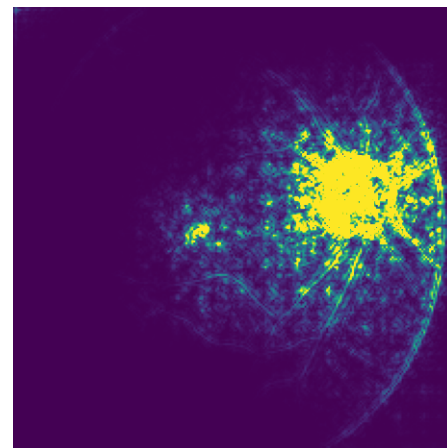

Supplement: S8 Fig — (ZIP) [file pone.0289211.s009.zip › figS8bii.pdf]
