# Supplementary material for: Learning from small data: Classifying sex from retinal images via deep learning
Source: PLoS One. 2023 Aug 3;18(8):e0289211. doi: 10.1371/journal.pone.0289211 (PMC10399793; doi:10.1371/journal.pone.0289211)
Supplement: Attachment — Submitted filename: review-response.pdf [file pone.0289211.s010.pdf]

# Authors' response to referees' reports on the paper *Learning from small data: Classifying sex from retinal images via deep learning*

By A. Berk, G. Ozturan, P. Delavari, D. Maberley, Ö. Yılmaz & I. Oruc

Submitted to *PLOS One*

We would first like to thank the two referees for carefully reading our manuscript and for their constructive criticism. We have incorporated the reviewers' comments and believe they have resulted in a substantial improvement in the quality of the manuscript.
Below, we include the reviewers' comments and our responses. We have left reviewer comments unchanged, with the exception of the formatting of the references mentioned by Reviewer 1.

## 1 Response to Reviewer 1

1. Title — few-shot learning is another research field of AI. The authors used more than 1000 fundus images to train the networks. In the literature, this volume has not been considered as "few examples".

   We agree that the literature for "learning from *few* examples" concerns learning from a significantly smaller number of examples (*e.g.,* $\lesssim 30$). We have revised the title of the manuscript to "Learning from small data:...", which we think better reflects the focus of our work.

2. The authors should read Noma et al. [1]. They found several patterns of fundus photography for determining sex. Very important paper in this field.

   Thank you for pointing us to this interesting work. From our reading, this work has two main contributions: a statistical analysis of manually selected features, and a ridge logistic regression analysis of those features. The statistical analysis was used to determine statistical significance of the feature-engineered characteristics (reported in [1, Table 1]). The logistic regression determined *feature importances* (reported on [1, p. 2784]). It is interesting to observe that the ovality ratio has a feature importance that is $\sim 34\times$ greater than the next largest feature importance. It is natural to interpret that the other features are therefore less distinguishing than ovality ratio. An interesting aspect of end-to-end deep learning approaches is the absence of manual feature engineering, combined with typically markedly greater efficacy. As the reviewer suggests, interpretability tools like Grad-CAM, occlusion maps, saliency maps, *etc.* can elucidate analogues of feature importances in the deep learning setting.

We have included references to this paper (without the above metacommentary) in the Introduction:

> Finally, interpretability is a major challenge for DL models. Though the model of Poplin et al. [2] can classify sex with high efficacy, it is challenging to determine the underlying elements of an image that determine the network's classification. For this reason, a subsequent work [1] used classical methods (manual feature engineering and logistic regression) to design an interpretable classifier (AUC = 61.5%).

Results (subsection 3.6):

> Notably, for this "Female" image, the mean activation is greater for the "Female" class label and tends to appear in more physiologically relevant regions of the fundus image (*i.e.,* about the optic disk and nearby vasculature; *cf.* [1, 3]).

and Discussion (subsection 4.4):

> We observed in Visual explanations for DOVS-ii classifiers that mean GGCAM behaviour for a matching class label (*e.g.,* "Female" image, "Female" class label) tends to have activation in image regions that have physiologically salient components (*e.g.,* vasculature and/or optic disk), some of which have previously been identified as potentially relevant markers for sex determination from fundus images [1].

3. The results of deep learning classifiers should be explained using Grad-CAM or occlusion maps. The authors should report the patterns of males and females. Additionally, comparison with other studies (Sex judgment using and Predicting sex from retinal fundus).

Model weights are needed for any such *ex post facto* interpretability analysis. Unfortunately, while model scores for each image remain available, the weights for the models in the manuscript are no longer available due to a catastrophic data loss incident. Grad-CAM and saliency maps are examined as an integral component of a subsequent work by a subset of the present authors [3], performed in the context of sex classification from retinal images by DL models using the ODIR data set. We have now included a reference to that work in our Introduction.

> An alternative approach retains the high-efficacy DL model, and creates *ex post facto* classifier interpretations using standard DL tools like class activation maps [4] or saliency maps [5]. Each is an integral component of a subsequent work by a subset of the present authors [3], in which a framework is developed for explainable classification of sex from

> fundus images by DL models.

Moreover, we have developed Grad-CAM images from new end-to-end DL models trained on the DOVS-ii dataset, following the same procedure as described for E1–E10. We have included validation and test scores for these models to show that they achieve similar performance to the previous DOVS-ii models (though it is natural to expect some variation due to stochasticity and change in training image resolution). There are several new sections that go into detail on the (in our opinion) quite interesting results: methods subsection 2.8 *Visual explanations for classification performance*; results subsection 3.6 *Visual explanations for DOVS-ii classifiers*; discussion subsection 4.4 *Visual explanations for (mis)classification*; and supplement subsection *S1 GGCAM plots*. These new subsection elements are marked with a "new" tag in the margin in lieu of blue highlighting.

4. Fig 1 — the concept of back-propagation is not necessary for the academic readers. Too basic.

Thank you for the suggestion. We have removed the figure.

5. According to the previous studies, it is well-known that deep learning can determine sex via fundus photography. Please highlight the novelty and academic contribution of this study.

The principal focus of the present manuscript to characterize the impact of small datasets in training deep learning models for complex classification tasks, specifically in the context of automated retinal image analysis. While large-scale approaches have shown great efficacy, few-shot learning methods have not yet been fully explored. We examine an intermediate regime between few shot learning and "big data" learning tasks. In particular, we examine efficacy of "simple" model training, performance on domain adaptation tasks and the effect of model ensembling. Thus we sketch a characterization of what can be expected from "classical" transfer-learning based deep learning approaches for realistic, intermediate data sizes, as well as a description of how these models might be expected to perform in the presence of new out-of-distribution data.
We have amended the final paragraph in the Introduction to better highlight this.

> In this study, we take a step toward examining this open question by developing and evaluating a DL model to classify patient sex from retinal images for small data set sizes. The DL model architecture is a state-of-the-art ResNet architecture, whose weights have been pre-trained on the ImageNet database. The pre-trained network is fine-tuned for classifying sex on a database of approximately 2 500 fundus images. Fur-

thermore, we investigate the stability of a neural network trained on a small database of images by testing its classification performance on out-of-distribution data. We approach this goal empirically through domain adaptation experiments, in which the performance of DL models trained on one dataset of retinal fundus images are then evaluated on another. Finally, we contrast the results of the models in this work with an enesemble model created by averaging scores of several individual models trained on small data sets.

6. Please clarify the demographic information of the participants. Age and ethnicity.

Ethnicity data are unavailable for both DOVS and ODIR datasets. Both the ODIR and the DOVS were composed of images taken for clinical purposes. In such contexts, ethnicity is not often included as a label, and furthermore, it is challenging to "infer" the ethnicity of a participant in the absence of a self report. Nevertheless, the ODIR images were acquired by the Shanggong Medical Technology Co., Ltd. from different hospitals and medical centers in China. Thus, overall, one can expect the patients to be predominantly East Asian [6]. The DOVS images were collected at VGH in Vancouver, thus it is plauisbly an ethnically diverse set, similar to Greater Vancouver Census demography over the years: approximately 43% European; 23% East Asian; 14% South Asian; 7% Southeast Asian; 3% Middle eastern; 2% Indigenous; 2% Latin American; 1.6% African. We have made a note regarding the (lack of) ethnicity information in Methods, omitting the speculative component of this remark.
Age information is already summarized in Figure 12 and Table 5 of the supplement. We have made two minor changes to the wording in Methods to better direct the inquiring reader.

7. Transfer learning or fine-tuning pretrained networks are not novel. The authors show what they did for few-shot or small-dataset-based learning. There are some few-shot learning articles Yoo et al. [7], Burlina et al. [8].

Thank you for pointing us to these articles. We agree that transfer learning is not new; in the originally submitted manuscript, we included the earliest explicit reference we found, from 2009 (see Section 2.3). We also include several citations to tutorials and surveys that provide, in our opinion, excellent further reading on transfer learning. The interesting works suggested by the reviewer examine transfer learning in two settings: rare disease classification from OCT images, and diabetic retinopathy classification from fundus images. These two settings are distinct from the setting considered in our work, which examines classification of sex from fundus images. We think the works highlighted by the reviewer serve as impetus to examine transfer learning for small data set DL classifiers on more challenging classification

problems (like sex classification), and so have included references to these works in our Introduction.

> An open challenge, crucial for widespread applicability, democratization, and generalization of DL in medical imaging is regarding whether high performance can be achieved with DL methods on challenging image classification tasks when the image database is small. Some work exists in this setting of so-called "low-shot" learning, but with different imaging modality (namely OCT) [7] or for a relatively easier classification task (namely DR) [8].

## 2 Response to Reviewer 2

The explanation are clear and understandable. All References are relevant. Representation of Pictorial statistical information can be made easier to understand. Overall the proposed work is good and accepted for publication.

We thank the reviewer for their feedback. It seems reasonable that our figures have the potential to be refined and we welcome specific feedback to improve the figures' presentation of our results. All the same, we think that our figures clearly communicate the elements we address in Discussion and so, in the absence of specific commentary from the reviewer, we have refrained from modifying them.

## References

[1] Saki Noma, Takehiro Yamashita, Ryo Asaoka, Hiroto Terasaki, Naoya Yoshihara, Naoko Kakiuchi, and Taiji Sakamoto. Sex judgment using color fundus parameters in elementary school students. *Graefe's Archive for Clinical and Experimental Ophthalmology*, 258(12): 2781–2789, 2020.

[2] Ryan Poplin, Avinash V Varadarajan, Katy Blumer, Yun Liu, Michael V McConnell, Greg S Corrado, Lily Peng, and Dale R Webster. Prediction of cardiovascular risk factors from retinal fundus photographs via deep learning. *Nature Biomedical Engineering*, 2(3):158, 2018.

[3] Parsa Delavari, Gulcenur Ozturan, Özgür Yılmaz, and Ipek Oruc. Artificial intelligence as a gateway to scientific discovery: Uncovering features in retinal fundus images. *arXiv preprint arXiv:2301.06675*, 2023.

[4] Ramprasaath R Selvaraju, Michael Cogswell, Abhishek Das, Ramakrishna Vedantam, Devi Parikh, and Dhruv Batra. Grad-CAM: Visual explanations from deep networks via gradient-

based localization. In *Proceedings of the IEEE international conference on computer vision*, pages 618–626, 2017.

[5] Karen Simonyan, Andrea Vedaldi, and Andrew Zisserman. Deep inside convolutional networks: Visualising image classification models and saliency maps. *arXiv preprint arXiv:1312.6034*, 2013.

[6] Shanggong Medical Technology Co. Ltd. Peking university international competition on ocular disease intelligent recognition. https://odir2019.grand-challenge.org, 2019.

[7] Tae Keun Yoo, Joon Yul Choi, and Hong Kyu Kim. Feasibility study to improve deep learning in OCT diagnosis of rare retinal diseases with few-shot classification. *Medical & biological engineering & computing*, 59(2):401–415, 2021.

[8] Philippe Burlina, William Paul, Philip Mathew, Neil Joshi, Katia D Pacheco, and Neil M Bressler. Low-shot deep learning of diabetic retinopathy with potential applications to address artificial intelligence bias in retinal diagnostics and rare ophthalmic diseases. *JAMA ophthalmology*, 138(10):1070–1077, 2020.
