# Supplementary material for: Learning from small data: Classifying sex from retinal images via deep learning
Source: PLoS One. 2023 Aug 3;18(8):e0289211. doi: 10.1371/journal.pone.0289211 (PMC10399793; doi:10.1371/journal.pone.0289211)
Supplement: Attachment — Submitted filename: response_r2.pdf [file pone.0289211.s011.pdf]

Authors' response (R2)

Learning from small data: Classifying sex from retinal images via deep learning

By A. Berk, G. Ozturan, P. Delavari, D. Maberley, Ö. Yılmaz, I. Oruc
Submitted to *PLOS One*

We thank the reviewers for their second round of careful attention to our manuscript, and note that all of their comments and requests have been adequately addressed. This except with regard to the inclusion of original fundus images (commented on by Reviewer 1): the original images in Figure 6 come from the UBC Ophthalmology Image database and its ethics do not allow us to publish these images.

Please note that we have checked and verified all references, particularly with regard to the retraction policy highlighted by the Academic Editor. We have done so using a combination of Zotero's automatic reference verification and manual search. We have moreover verified that no preprints in our bibliographic matter have since been published. We have determined all bibliographic information to be complete and correct.
